# Cannabinoids and Cannabinoid Receptors: The Story so Far

**DOI:** 10.1016/j.isci.2020.101301

**Published:** 2020-06-20

**Authors:** Fred Shahbazi, Victoria Grandi, Abhinandan Banerjee, John F. Trant

**Affiliations:** 1 Department of Chemistry and Biochemistry, University of Windsor, Windsor, ON N9B 3P4, Canada

**Keywords:** Medical Substance, Supramolecular Chemistry, Molecular Biology, Structural Biology

## Abstract

Like most modern molecular biology and natural product chemistry, understanding cannabinoid pharmacology centers around molecular interactions, in this case, between the cannabinoids and their putative targets, the G-protein coupled receptors (GPCRs) cannabinoid receptor 1 (CB_1_) and cannabinoid receptor 2 (CB_2_). Understanding the complex structure and interplay between the partners in this molecular dance is required to understand the mechanism of action of synthetic, endogenous, and phytochemical cannabinoids. This review, with 91 references, surveys our understanding of the structural biology of the cannabinoids and their target receptors including both a critical comparison of the extant crystal structures and the computationally derived homology models, as well as an in-depth discussion about the binding modes of the major cannabinoids. The aim is to assist in situating structural biochemists, synthetic chemists, and molecular biologists who are new to the field of cannabis research.

## Highlights

• Cannabinoid research has greatly expanded
• Structural biology and computational chemistry jointly provide mechanistic insight
• Structural data are being generated at an exponentially increasing rate
• Phytocannabinoid targeting of other GPCR receptors deserves investigation

## Graphical Abstract

**Figure:**
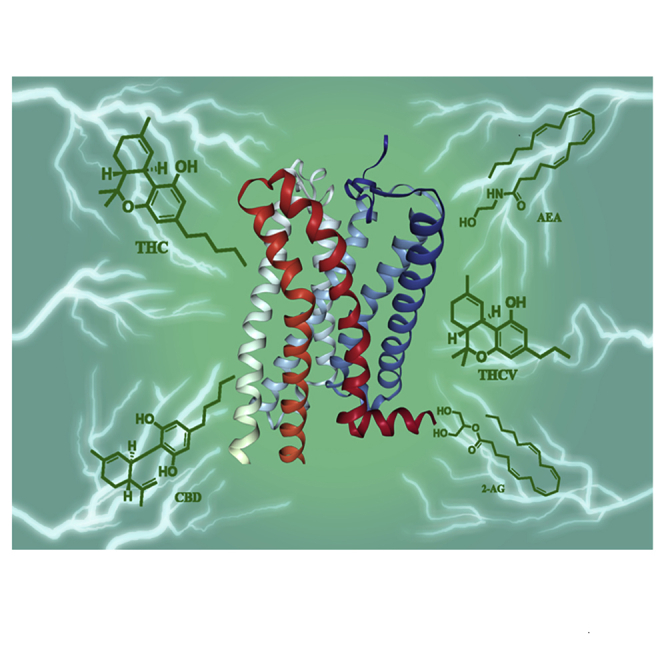

## Introduction

Despite its reputation, with the exception of Δ^9^-tetrahydrocannabinol (**THC**), the phytocannabinoids produced by *Cannabis sativa* are minimally psychoactive; however, they constitute a potentially rich source of therapeutics owing to their interaction with central nervous system (CNS) receptors (Pertwee, 2006). **THC** and cannabidiol (**CBD)** are by far the most abundant. **THC** was discovered in 1964, whereas the non-psychoactive **CBD** was discovered in 1942 (Reekie et al., 2017). Many of cannabis' proposed medical benefits are thought to arise from **CBD**; however, recent clinical and scientific work suggests that the minor cannabinoids also play an important role (Turner et al., 2017). Understanding the biological activity of these minor components will guide plant breeding to develop new cultivars designed with particular medical applications in mind.

**Figure 1:**
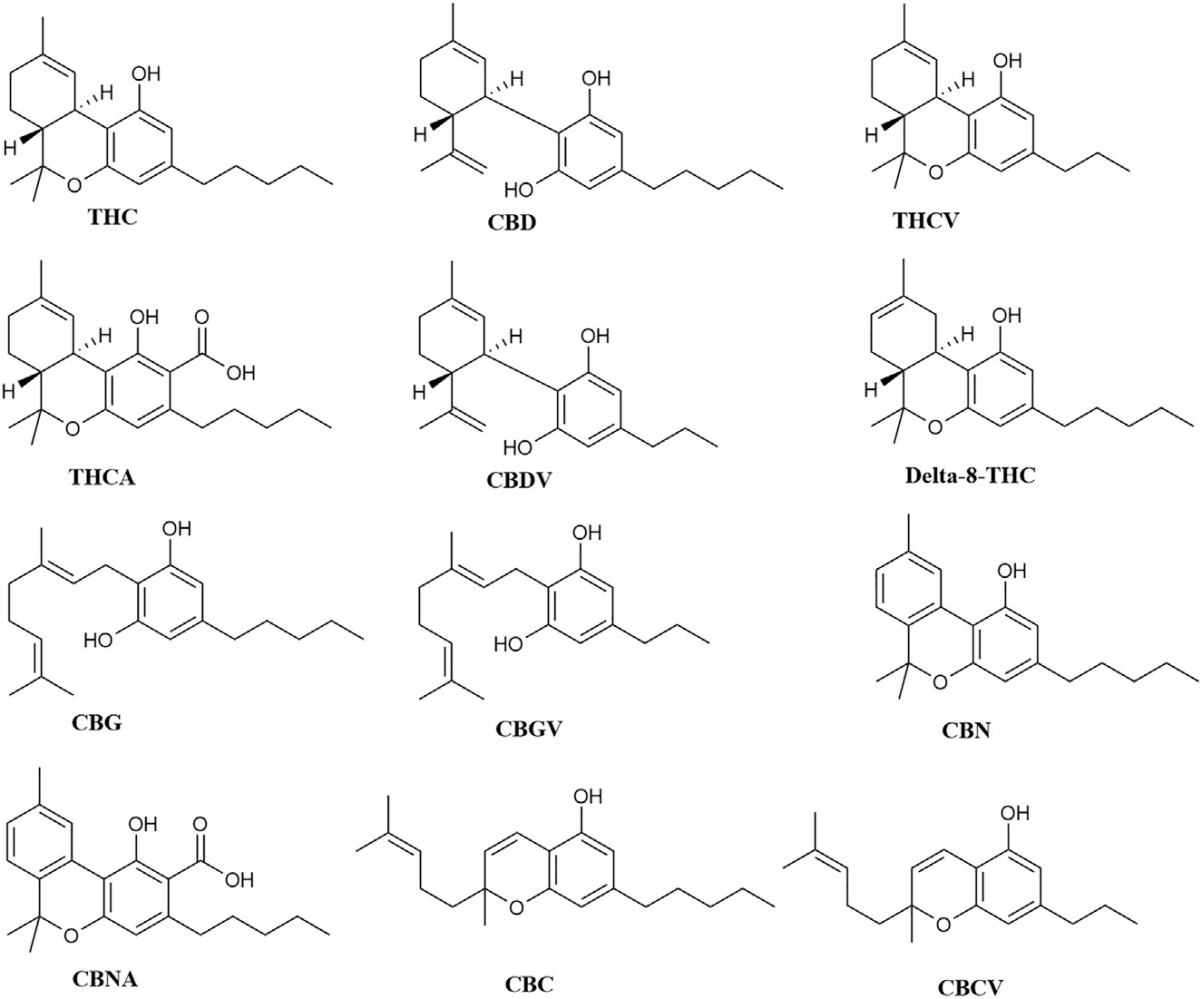
Structure of Cannabinoids Archetypal of Their Structural Families

As of 2020, over 150 minor cannabinoids, collectively constituting less than 1% of the total dry mass of cannabis bud, have been identified (Citti et al., 2019a). Cannabinoids isolated from *cannabis sativa* include tetrahydrocannabivarin (**THCV**) (Gill, 1971), Δ^9^-tetrahydrocannabinolic acid (**Δ**^**9**^**-THCa**) (Rosenqvist and Ottersen, 1975), cannabidivarin (**CBDV**) (Vollner et al., 1969), Δ^8^-tetrahydrocannabinol (**Δ**^**8**^**–THC**) (Fahrenholtz et al., 1966), cannabigerol (**CBG**) (El-Darawy et al., 1972), cannabigerovarin (**CBGV**) (Shoyama et al., 1975), cannabinol (**CBN**) (El-Darawy et al., 1972), cannabinolic acid (**CBNa**) (Shoyama et al., 1970), cannabichromene (**CBC**) (El-Darawy et al., 1972), and cannabichromevarin (**CBCV**) (Shoyama et al., 1975) (Figure 1). Most of these have been understudied; extant articles normally only describe their isolation and structure determination: their biological activity remains unknown. Two human endocannabinoids have also been identified: anandamide (**AEA**) from brain tissue and 2-arachidonoyl glycerol (**2-AG**) from peripheral tissue (Reggio, 2010; Devane et al., 1992; Stella et al., 1997). The primary targets of the cannabinoids are thought to be cannabinoid receptors 1 (CB_1_) and 2 (CB_2_), which are class A G-protein coupled receptors (GCPRs). However, the structural similarity of GPCRs and the structural diversity of the phytocannabinoids, coupled with the anecdotal and preliminary medical studies into the physiological effects of cannabis, hint at a broader scope of interaction.

Exploring this exciting new landscape requires a thorough understanding of the known molecular interactions between cannabinoids and receptors; consequently, this review summarizes our existing knowledge of the interactions between the cannabinoids and the two cannabinoid receptors, CB_1_ and CB_2_, based both on structural biology and associated computational studies. As the first synthesis of the literature in this field, we believe that it will prove as useful to the community as it has to us to better explore the molecular pharmacology of these fascinating molecules. The target audience is natural product, computational and synthetic chemists, as well as structural biologists and protein crystallographers seeking a background on this field.

### General Structure, Physiological Role, and Mechanism of Activation of GPCRs

**Figure 2:**
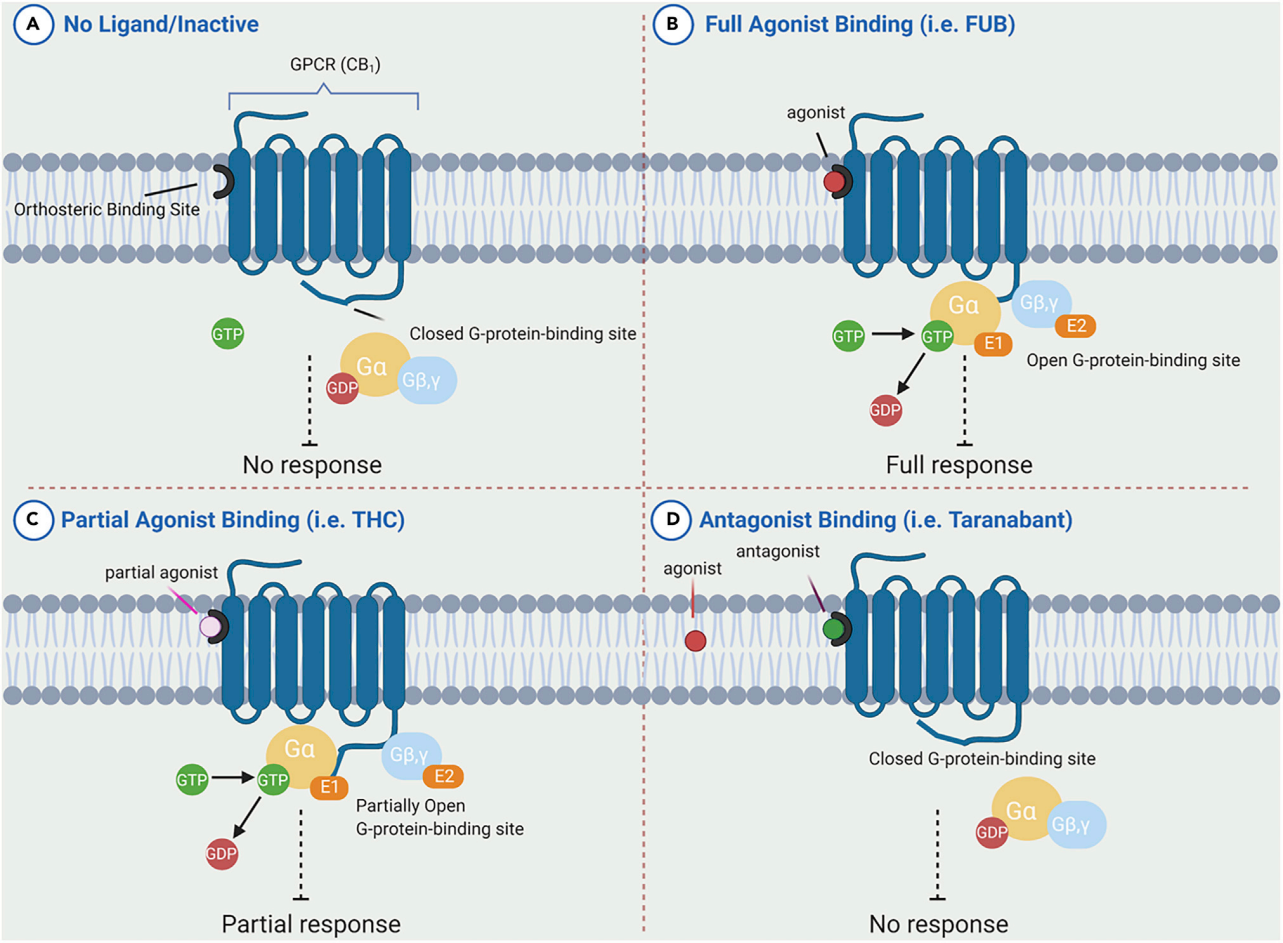
Schematic Representation of Signal Transduction by Ligand Interactions with the GPCRs The state of the receptor in each panel is described therein.

GPCRs are a diverse family of eukaryote-specific membrane receptors that translate external signals, such as light, peptides, lipids, sugars, and proteins, into specific cellular responses. Their centrality to cellular signaling has made them arguably the central focus of modern drug discovery (Hauser et al., 2017). GPCR domains comprise the extracellular N terminus, seven transmembrane alpha helices (TM), loops connecting the TMs, and an intracellular C terminus. Ligand binding generally occurs within a binding site gap formed by the TM bundle, directly to a pocket formed by the extracellular loops, or to a combination of extracellular loop and binding site gap residues. Binding induces a conformational change in the receptor, causing activation of a G protein docked to the internal face (Figure 2), which then initiates a specific cellular process (Latorraca et al., 2017; Weis and Kobilka, 2014). In general, an agonist-bound receptor activates an appropriate G protein that promotes dissociation of GDP (Figure 2) (Tuteja, 2009). GPCR ligands fall into four categories depending on the nature of their interaction: agonists, antagonists, partial agonists, and inverse agonists. Agonists bind to the receptor and elicit a cellular response by causing a conformational change. Antagonists bind, prevent agonists from binding, and do not elicit any response. A partial agonist is an intermediate class that upon binding does not invoke the complete agonist conformational change, but still allows for partial activity; simultaneously, they “block” the receptor from being available for full agonist binding. When both a full agonist and partial agonist are present, the partial agonist acts as a competitive antagonist, producing a net decrease in the receptor's activation. Inverse agonists bind to a receptor but induce a physiological response opposite to what would be expected from an agonist. The affinity of a ligand for the receptor is independent of the role: weakly binding full agonists and strongly binding partial agonists are both known. Biophysical studies indicate that ligand-induced receptor activation generally proceeds by changing the relative orientations of TM3 and TM6 (Nakanishi et al., 2006), with the intracellular end of TM6 moving away from TM3 by hinging and moving “up” toward the membrane (Jensen et al., 2001). This modification then affects the conformation of the G protein-interacting intracellular loops of the receptor and thus uncovers previously masked G protein-binding sites. Upon binding of extracellular ligands such as phytocannabinoids, GPCRs interact with a specific subset of heterotrimeric G proteins that can then, in their activated forms, inhibit or activate various effector enzymes and/or ion channels. Molecular cloning studies have shown that GPCRs form one of the largest protein families found in nature, and it is estimated that approximately 1,000 different such receptors exist in mammals; of these, our focus in this review is limited to two, CB_1_ and CB_2_: the cannabinoid receptors (Wess, 1997), but it is likely that cannabinoids can also interact with other members of the protein family.

### A Structural Tour of Cannabinoid Receptors 1 and 2

The human CB_1_ and CB_2_ receptors are closely related GPCRs, exhibiting approximately 44% amino acid similarity overall and 68% homology in the TMs (Munro et al., 1993; Hryhorowicz et al., 2019). Molecular dynamic studies indicate that ligand binding to both CB_1_ and CB_2_ occurs through lateral insertion via the lipid bilayer rather than directly from solution (Reggio, 2010). The most important sequence differences between CB_1_ and CB_2_ are in the N-terminal extracellular loop II (ECL2) involved in cannabinoid binding (Shao et al., 2016), the C-terminal sequence of TM7, and the internal C terminus itself (Montero et al., 2005). The other key feature in CB receptor is the presence of a toggle switch, whose activation leads to G protein binding: in CB_1_ the twin toggle switch involves two residues, F200 and W356 on TM3 and TM6, respectively; in contrast, CB_2_ has a single toggle switch residue, W258, on TM6. Changing their relative position opens the two helices like chopsticks revealing the G_*i*_ protein binding site. Determining their status defines whether a ligand is an agonist or antagonist (McAllister et al., 2004). These structural differences define ligand preference: CB_1_ requires the polycyclic core of the potential ligand to have a C_3_ alkyl chain of five or more carbons, whereas CB_2_ recognizes smaller classical cannabinoids (Figure 1). Similarly, etherification at C_1_ leads to CB_2_-selective compounds. As CB crystal structures have only very recently been reported, historical, pre-2016, structural work has largely involved homology modeling based on the rhodopsin crystal structure (PDB: *1F88*), (Palczewski et al., 2000), which shares 21% and 20% homology with CB_1_ and CB_2_, respectively*.*

### Cannabinoid Receptor 1 (CB_1_)

CB_1_ receptors, ubiquitous in the CNS, are most highly expressed by the axons and presynaptic termini of neurons in the amygdala, hippocampus, cortex, basal ganglia outflow tracts, and cerebellum (Baker et al., 2003). Strongly associated with GABAergic and glutamergic cells, their activation inhibits GABA and glutamate release, respectively (Baker et al., 2003). CB_1_ receptor activation has been found to increase potassium and calcium ion channel activity; consequently, CB_1_ modulates neurotransmitter release in a dose-dependent and pertussis toxin-sensitive manner. The receptor can exist as a homodimer, or as a heterodimer or hetero-oligomer complexed with other GPCRs. In addition to the main binding site, the CB_1_ receptor also possesses an allosteric modulatory binding pocket.

### Crystal Structure of CB_1_

**Figure 3:**
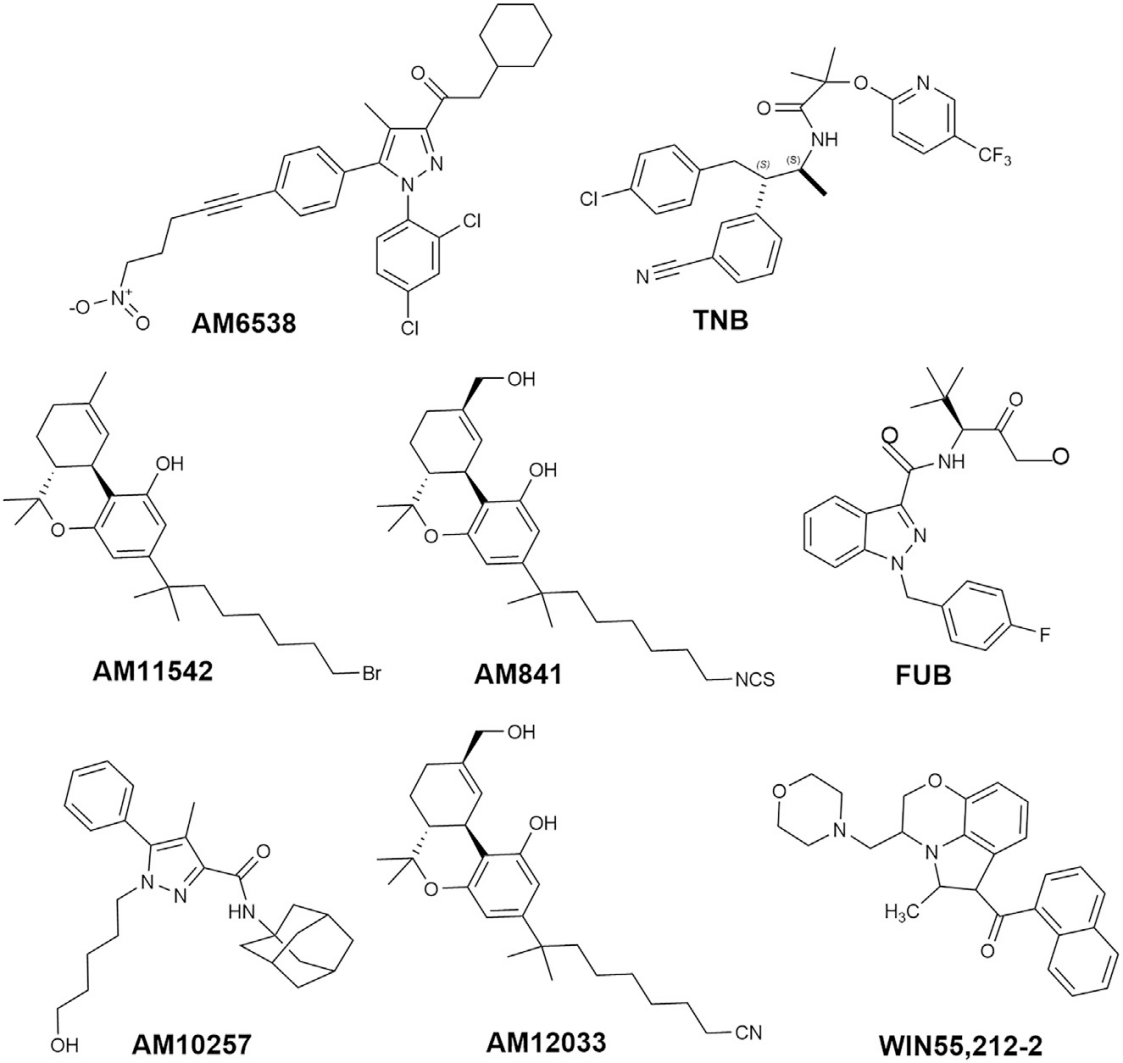
Structure of Synthetic Analogs AM6538, TNB, AM11542, FUB, AM10257, AM12033, and WIN55,212-2

Four crystal structures and two cryoelectron microscopic (cryo-EM) structures of CB_1_ are available with synthetic cannabinoids (Figure 3). Four are co-crystallized with a ligand: one bound to antagonist **AM6538**, one bound to an inverse agonist taranabant (**TNB**) (Hua *et al.;* Shao et al., 2016), two bound to a full agonist (**AM11542** and **AM841**) (Hua et al., 2017), most recently, two Cryo-EM structure: bound to full agonist with the G_*i*_ protein bound (MDMB-Fubinaca, **FUB**) (Krishna Kumar et al., 2019), and **AM841** (Hua et al., 2020). Crystallization in the presence of a phytocannabinoid remains elusive.

**Figure 4:**
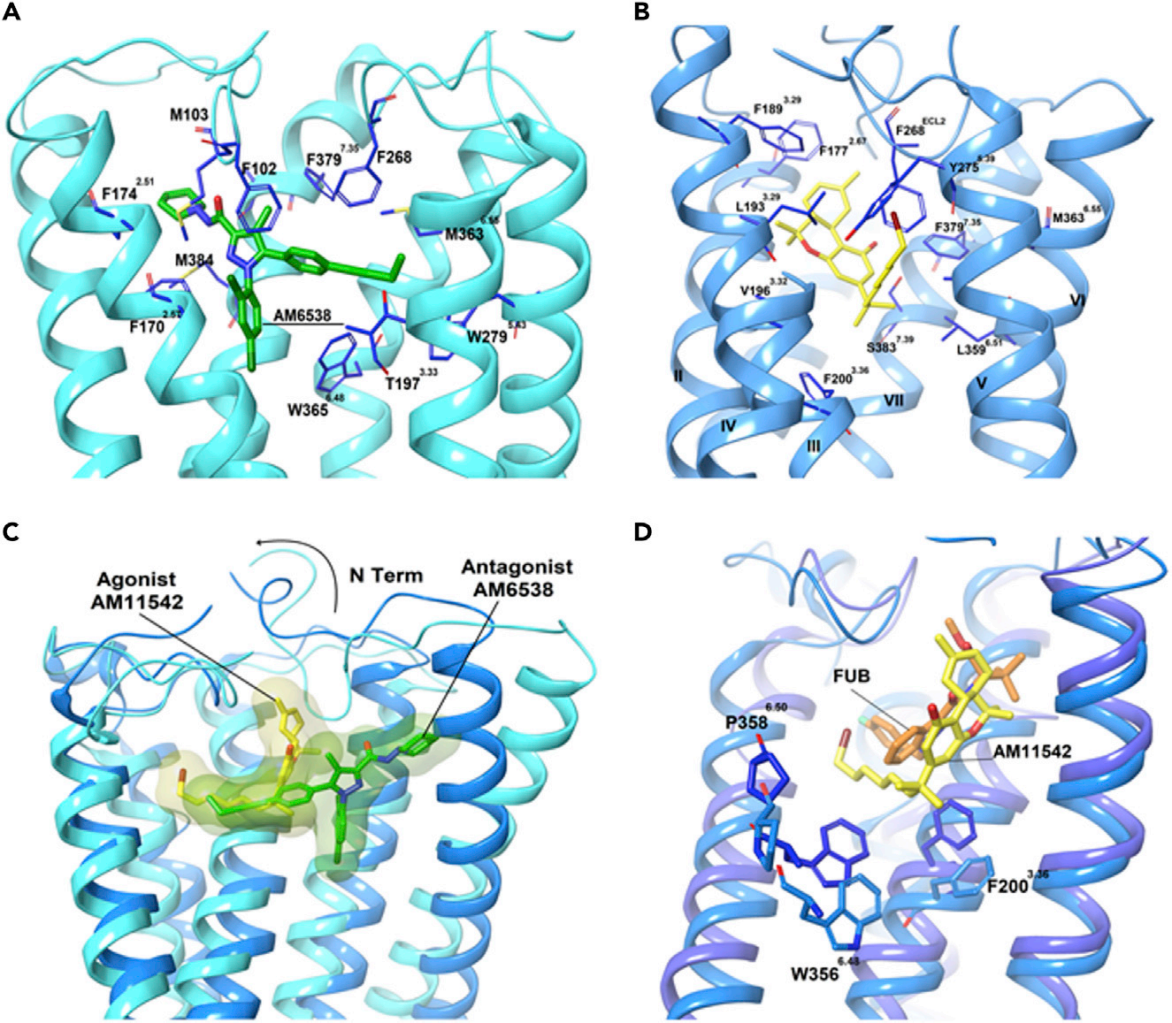
Comparison of CB1 Binding to Different Ligands Analysis of the ligand binding pocket (A) **AM6538@CB**_**1**_ (*5TGZ*) and (B) **AM11542@CB**_**1**_ (*5XRA*). (C) Comparison of the agonist-bound (blue) and antagonist-bound (light blue) CB_1_. (D) Superposition of **FUB@CB**_**1**_ (navy) and **AM11542@CB**_**1**_ (blue). Data used to prepare the figures were obtained from Hua et al. (2016), Figures 4A; Hua et al. (2017), Figure 4B; and Krishna Kumar et al. (2019), Figure 4D.

The first crystal structure of CB_1_ was reported by Liu and coworkers in 2016 as a complex with antagonist **AM6538 (AM6538@CB**_**1**_, PDB: *5TGZ*, Figure 4A, Video S1) (Hua et al., 2016).The authors noted that the binding pocket of CB_1_ is quite plastic, as is typical for class A GPCRs, explaining the broad substrate tolerance of the receptor. In the same year, Shao et al. used GPCR engineering and lipidic cubic phase crystallization to determine the structure of the human CB_1_ receptor bound to antagonist **TNB** (**TNB@CB**_**1**_, PDB: *5U09*, Video S1) (Shao et al., 2016). The conformational flexibility of the ligand binding site is greater in **AM6538@CB**_**1**_ than in **TNB@CB**_**1**_, leading to a more disordered structure in the former, whereas **TNB@CB**_**1**_ has higher electron densities around the ligand and the critical N-terminal domain providing more specific structural detail; this is supported by the high B factors in the refined model for **AM6538@CB**_**1**_ (average B = 134.3 Å^2^ for residues 99–112 and B = 119.5 Å^2^ for the ligand), whereas the B factors for **TNB@CB**_**1**_ are far lower (average B = 61.7 Å^2^ for residues 100–112 and B = 42.0 Å^2^ for the ligand) (Shao et al., 2016). However, both structures remain quite similar in gross morphology providing mutual confidence in the structures and highlight the CB_1_-specific feature: the extracellular surface, including the highly conserved membrane-proximal N-terminal region differs considerably in conformation from other lipid-activated GPCRs. In **TNB@CB**_**1,**_ the ECL2 and the membrane-proximal N-terminal sequence cooperate to construct a lid over the orthosteric pocket, essentially isolating the ligand from the solvent. **TNB** is tightly held in the pocket, making multiple contacts with both TM1 and TM7 at the TM1–TM7 opening, blocking the entry of endocannabinoids that prefer to sit deeper in the pocket. This is an unusual feature in a GPCR.

Video S1. Crystal Structures of AM6538@CB1 (*5TGZ*), TNB@CB1 (*5XRA*), AM11542@CB1, and FUB@CB1 (*6N4B*) Animation of Figure 4 to provide different points of view for the reader; analysis of the ligand binding pocket (A) **AM6538@CB**_**1**_ (*5TGZ*) and (B) **AM11542@CB**_**1**_ (*5XRA*). (C) Comparison of the agonist-bound (blue) and antagonist-bound (light blue) CB_1_. (D) Superposition of **FUB@CB**_**1**_ (navy) and **AM11542@CB**_**1**_ (blue).1

The first agonist-bound CB_1_ crystal structure was reported in 2017 (**AM11542@CB**_**1**_ PDB: *5XRA*) (Figure 4B, Video S1) (Hua et al., 2020). This structure was largely consistent with that identified by Kobilka et al. in 2019 with the highly potent agonist **FUB** (**FUB@CB**_**1**_ PDB: *6N4B*, Video S1) (Krishna Kumar et al., 2019). Compared with the antagonists, the extracellular domains of TM1 and TM2 of CB_1_ move inward and the intracellular part of TM6 moves outward in the active vis-à-vis the inactive state. These motions shrink the volume of the orthosteric ligand-binding site by 53%, open the twin toggle switch comprising F200 and W356, and correspondingly increase the surface area of the G-protein-binding region of the receptor, activating the GPCR. In **AM11542@CB**_**1**_, the N terminus sits over the ligand-binding pocket and is not directly involved in agonist binding; this differs from the V-shaped loop it adopts in the **AM6538@CB**_**1**_ structure where it caps the binding site (Figure 4C). The C_3_ alkyl chain of **AM11542** occupies a similar position as that of antagonist **AM6538@CB**_**1**_, indicating that the binding differences between agonists and antagonists is very subtle. The most notable conformational change occurs for TM1 and TM2. In **AM11542@CB**_**1**_, TM1's extracellular sequence bends inward by 6.6 Å, and TM2 also rotates inward by 6.8 Å. In **FUB@CB**_**1,**_ TM2's rotation repositions F170^2.5^, F174^2.61^, F177^2.64^, and H178^2.65^, orienting them toward the pocket in the active conformation to interact with the agonist. **FUB** association stabilizes CB_1_ in an active conformation by forcing open the “toggle twin switch” F200^3.36^ and W356^6.48^ residues. The shifting of W356^6.48^ leads to the relaxation of the kink at P358^6.50^ (Figure 4D), straightening out TM6, which opens up the cytoplasmic binding site allowing the C-terminal α_5_ helix of G_*I*_ to bind to the GPCR and initiate the internal signaling cascade. **FUB**'s rigidity in forcing these changes makes it a full agonist compared with the more flexible partial agonists, like **THC**, that provide for greater receptor flexibility, allowing it to not be forced into the open state: the critical H-bond and π – π interactions between the ligands and CB_1_ that drive these changes are provided in Table 1.

**Table 1:** H-Bond and π – π Interactions of Ligands of All Crystal Structures of CB_1_ and CB_2_

			CB1				CB2	
PDB Code	*5XR8*	*5XRA*	*6N4B*	*5TGZ*	*5U09*	*6KPC*	*6PT0*	*5ZTY*
Ligand	**AM841**	**AM11542**	**FUB**	**AM6538**	**Taranabant**	**AM12033**	**WIN 55,212-2**	**AM10257**
Binding affinity Ki (nM)	1.14	0.11	0.098	0.038	0.13	0.37	3.3	0.61
Hydrogen bond	S383^7.39^	S383^7.39^	S383^7.39^			Ser285^7.39^		
	I267^ECL2^		H178^2.65^					S165^4.57^
	Y275^5.39^							
π – π interaction			W279^5.43^			F94^2.64^	F94^2.64^	
	F170^2.57^	F170^2.57^	F170^2.57^	F170^2.57^		F183^ECL2^		F183^ECL2^
	F268^ECL2^	F268^ECL2^	F268^ECL2^	F268^ECL2^		F281^7.35^		
	F379^7.35^	F379^7.35^		F102^N-term^			F91^2.61^	
	F189^3.25^	F189^3.25^		W356^6.48^			F117^3.36^	F117^3.36^
	F177^2.64^	F177^2.64^					W258^6.48^	W258^6.48^
Reference	(Hua et al., 2017)	(Hua et al., 2017)	(Krishna Kumar et al., 2019)	(Hua et al., 2016)	(Shao et al., 2016)	(Hua et al., 2020)	(Xing et al., 2020)	(Li et al., 2019)

### Cannabinoid Receptor 2

The CB_2_ receptor is closely related to the CB_1_ receptor (vide supra), with seven transmembrane helices, a glycosylated N terminus, and the C-terminal helix embedded in the cellular matrix. The discovery in 1993 of CB_2_ receptors provided a partial explanation for the immunomodulatory properties of cannabinoids (Munro et al., 1993). Expressed mainly on immune system cells and the astrocytes and microglia in the CNS (Mackie, 2008), CB_2_ activation is associated with neurodefense functions and ensuring maintenance of bone mass and the reduction of inflammation. CB_2_ agonists have been explored for slowing neurodegenerative disorders such as Huntingtons chorea and Alzheimer disease (Maccarrone et al., 2007). CB_2_ receptors function by inhibiting the activity of adenylyl cyclase through their G_*I*_ /G_*oα*_ subunits (Bouaboula et al., 1996; Soethoudt et al., 2017) by coupling to stimulatory Gα_i/o_ subunits, causing a rise in intracellular cAMP levels, and by influencing the Ras-Ref-MEK-ERK pathway, which has a profound effect on mature and neoteric tissues (Slipetz et al., 1995; Saroz et al., 2019).

### Crystal Structure of CB_2_

The first crystal structure of CB_2_, bound to high-affinity synthetic antagonist **AM10257,** was reported by Hua and coworkers in 2019 (PDB:*5ZTY*, Figure 3, Video S2) (Li et al., 2019).The C-terminal intracellular helix adopts an inactive conformation similar to that of antagonist-bound CB_1_; the extracellular moieties of antagonist-bound CB_2_ and CB_1_ differ, most notably within TM1 and TM2. Unlike its adoption of a V-shaped loop for direct involvement in ligand binding in antagonized CB_1_, the N-terminal helix of **AM10257@CB**_**2**_ sits over the orthosteric pocket with no direct involvement in antagonist binding; however, similar to CB_1_, ECL2 in CB_2_ is stabilized by an internal disulfide bond (C174–C179) maintaining the key conformational lock holding the binding site in the ligand-binding conformation.

Video S2. Crystal structures of AM10257@CB2 (*5ZTY*), AM12033@CB2 (*6KPC*), and WIN55212-2@CB2 (*6PT0*) Animation of Figure 8 to provide different points of view for the reader. (A) Chemical structures and predicted binding poses of **THC** with inactive structure, (A) PBD: *5TGZ* and (B) PDB: *5U09* (**15**) (transparent magenta sticks), and (C) with active structure PDB: *5XRA*. **THC** (pink) and **TNB** (light green).2

**Figure 5:**
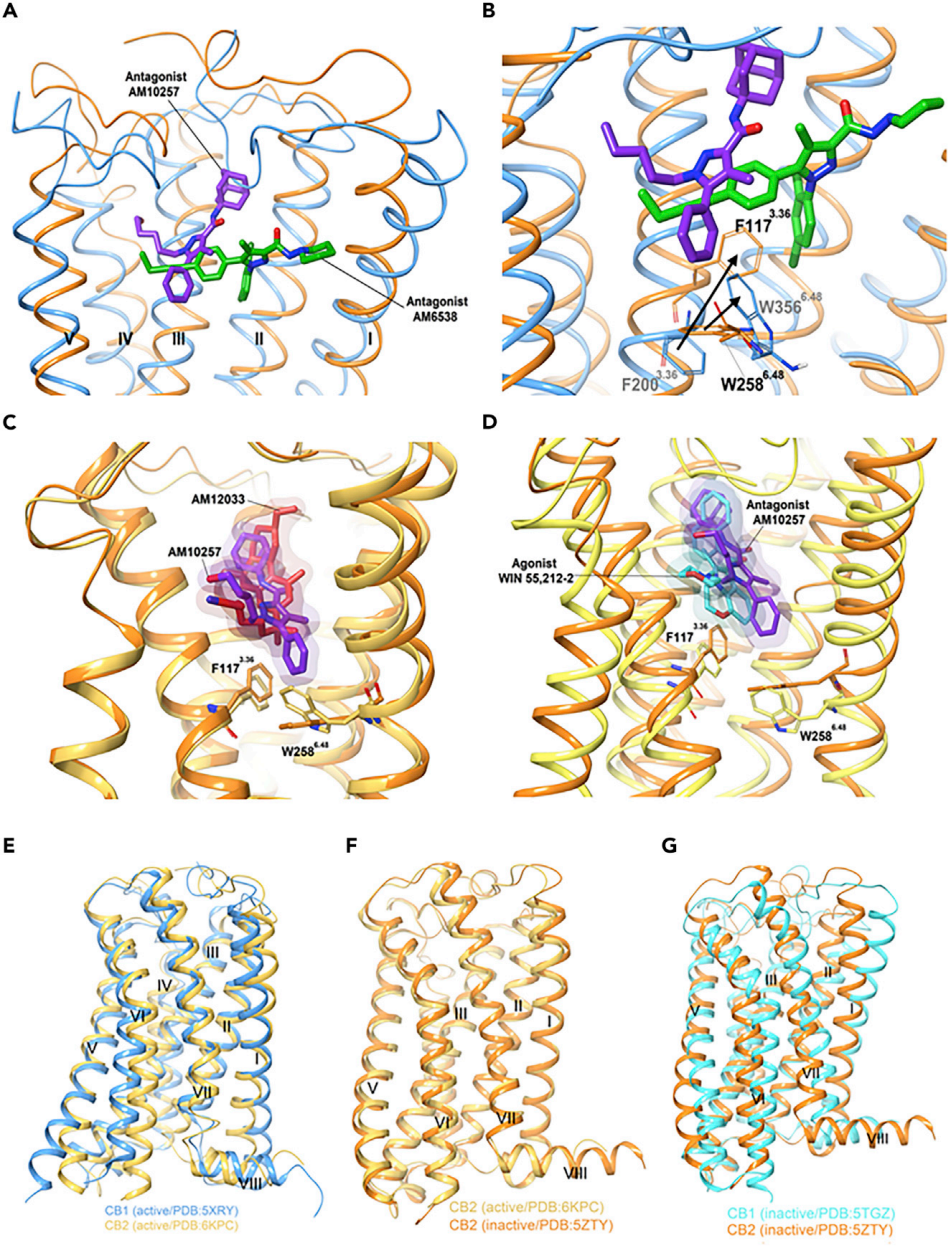
Comparison of the Ligand-Binding Modes of CB_1_ and CB_2_ (A and B) (A) Superposition of **AM6538@CB**_**1**_ (*5TGZ*) (blue) and **AM10257@CB**_**2**_ (*5ZTY*) (orange) ligand-binding pockets and (B) conformational difference of F200 and W258 in antagonist bound CB_2_ and CB_1_. (C) Comparison of the “toggle switch” residue conformation in agonist **AM12033@CB**_**2**_ (*6KPC*) (gold) and **AM10257@CB**_**2**_ (*5ZTY*) (orange). (D–G) (D) **WIN55212-2@CB**_**2**_ (*6PT0*) (yellow) orthosteric pocket shows the direct contact of the ligand with residues F117^3.36^ and W258^6.48^ compared with **AM10257@CB**_**2**_ (orange). Comparison of the gross morphology of the receptors: (E) active CB_1_ and CB_2_ (F), active CB_1_ and inactive CB_2_, and (G) inactive CB_1_ and CB_2_. Data used to prepare the figures were obtained from Li et al. (2019), Figures 5A and 5B; Hua et al. (2020), Figures 5C and 5E–5G; and Xing et al. (2020), Figure 5D.

There are major structural differences between the two ligand-bound receptors, despite the structural similarity of **AM10257** and **AM6538**. Instead of adopting the extended conformation of **AM6538** in **AM6538@CB**_**1**_, **AM10257** assumes a more constrained binding pose in **AM10257@CB**_**2**_. In **AM10257@CB**_**2**_, the ligand forces TM3 and TM4 over by 6.1 Å relative to **AM6538@CB**_**1**_**,** using the pyrazole ring as a common reference point (Figure 5A). Similarly, the extracellular moieties of TMI and TM2 collapse toward the ligand binding site allowing F87^2.57^ and F91^2.61^, to form hydrophobic interactions with **AM10257**. In contrast to the horizontally extended arm of **AM6538** in **AM6538@CB**_**1**_, that of **AM10257** in **AM10257@CB**_**2**_ assumes a nearly vertical pose. This likely all relates to the differential behavior of the N-terminal sequence in the two receptors: in CB_1_ the adopted V-shaped loop inserts into the cannabinoid binding pocket, whereas in CB_2_ it remains extended and does not interact with the pocket. The other main difference in antagonist-bound CB_2_ and CB_1_ structures is the toggle switch residue W258^6.48^. **AM10257** confines the side chain of W258^6.48^ limiting the outward movement of TM6, whereas in **AM6538@CB**_**1**_ the ligand drives TM1 and TM2 apart. F200 functions as a latch to restrict the movement of W356 and locks CB_1_ in the inactive state (Figure 5B). Computational chemistry has also contributed to our understanding of the receptor. Docking studies involving structurally related CB_2_ agonist **MRI2594**, and antagonist **MRI2687** (Ogawa et al., 2015), which differ only by the length of one side chain, demonstrate the mechanism of activation of the toggle switch residue W258^6.48^. W258 is a highly conserved residue in class A GPCRs and has been reported to have a crucial role in GPCR activation (Lin and Sakmar, 1996). This observation not only supports the identification of W258^6.48^ as a key residue but also demonstrates the very fine line between the order of interaction involved in the binding of CB_2_ agonists and antagonists. The antagonist-binding motif in CB_2_ resembles the agonist-binding motif in CB_1_, especially in the conformations of the N termini. In addition, the volume of the CB_2_-antagonist binding pocket (447 Å^3^) is closer to that of activated **AM11542@CB**_**1**_ (384 Å^3^) than to that of antagonized **AM6538@CB**_**1**_ (822 Å^3^). This provides the structural basis for the observation that CB_2_ antagonists are often CB_1_ agonists (Ogawa et al., 2015), highlighting the yin-yang relationship between CB_1_ and CB_2_.

During the preparation of this review, Hua and colleagues reported a crystal structure of the agonist **AM12033** (Figure 3, Video S2)-bound CB_2_, obtained from X-ray diffraction data (**PDB**: *6KPC*).(Hua et al., 2020) It was noted that the main interactions in **AM12033**@CB_2_ are hydrophobic and aromatic in nature (Table 1). The binding pockets associated with agonist **AM12033** and antagonist **AM10257** were largely similar, but the inward movements of extracellular parts of TM1, TM4, and TM7 lead to the agonist's binding pocket being more compact. The W258^6.48^ “toggle switch” experiences an especially strong shift in **AM12033@CB**_**2**_ (Figure 5C) (Hua et al., 2020).

Xing and co-workers reported the electron microscopic structure of CB_2_ bound to a more potent agonist, **WIN55,212-2** (Figure 3; **PDB:** *6PT0*, Video S2) (Xing et al., 2020). Although this structure shows near identity to the **AM12033@ CB**_**2**,_ there were subtle differences: **AM10257** penetrates deeper into the binding pocket (*ca.* 2.8 Å) compared with **WIN55,212-2,** resulting in different conformations for toggle switch W258^6.48^. The slight rotation of F117^3.36^ permits W258^6.48^ to interact only weakly with **WIN 55,212-2**, forcing it 1.2 Å further away from the ligand than in the case of **AM10257** (Figure 5D). The consequent outward movement of the cytoplasmic end of TM6 further widens the G protein binding site. Even minor changes arising from the steric effects of CB_2_ ligands on W258^6.48^ play a crucial role in determining the relative agonism of CB_2_ ligands (Figure 5D).

Although **AM841@CB**_**1**_ and **AM12033@CB**_**2**_ are similar, the receptors exhibit distinct characters in terms of ligand and G protein selectivity and activation process (Figures 5E–5G). The single residue difference in ICL-2 (L222 in CB_1_ and P139 in CB_2_) may contribute to the G protein coupling diversity of the cannabinoid receptors; whereas CB_2_ is G_*i*_ specific, CB_1_ can bind other G proteins such as G_*s*_ (G protein-stimulating adenylate cyclase), and G_*q*_ (G protein-activating phospholipase C and G protein-increasing cytosolic Ca^2+^). The high degree of structural similarity in the orthosteric binding pockets between agonist-bound CB_2_ and CB_1_ imposes substantial challenges for receptor-selective agonist design.

### Homology Modeling

As discussed, it is only very recently that X-ray and cryo-EM-derived structures of the cannabinoid receptors have been available for analysis. Lacking these models, various computational techniques such as 3-dimensional quantitative structure-activity relationship (3D-QSAR), pharmacophore mapping, homology modeling, docking, and virtual screening techniques have been used extensively to derive information about the CB receptors from the crystal structures of related proteins, such as rhodopsin. The first three-dimensional model of a human cannabinoid receptor was constructed by Mahmoudian using bacteriorhodopsin as the structural template, with the aid of the SYBYL and MOPAC packages in 1997 (Mahmoudian, 1997). **THC** was then docked into the internal cavity utilizing AutoDock. This example shows the challenges in this type of work: the software and computing power 23 years ago was greatly inferior to modern tools, and the available crystal structure of the base protein, bacterial rhodopsin, was not even a GPCR protein leading to unavoidable error. However, it was the best that could be done at the time, and some of his findings, such as the criticality of the phenolic hydroxyl of **THC** for CB_1_ binding, have been validated by later structure-activity relationship (SAR) studies (Huffman et al., 1996).

In 1998, Shire and colleagues (Rinaldi-Carmona et al., 1998) used the WHATIF (Vriend, 1990) program for sequence manipulations and model energy refinement and SYBYL modeling packages based on the 1998 refined crystal structure of GPCR bovine rhodopsin (**PDB**: *1F88*) (Krebs et al., 1998). The challenge in homology modeling without a crystal structure is in validating the result. Consequently, it can be useful to generate multiple versions from different base proteins. In this vein, Latek and coworkers developed structures of both the CB_1_ and CB_2_ receptors based on the β2-adrenergic receptor (**PDB**: *3D4S*) (Latek et al., 2011) and compared the result with both rhodopsin-based models. All three models predicted the rough morphology of the orthosteric pocket, and have been broadly validated by crystallography (Durdagi et al., 2010).

**Figure 6:**
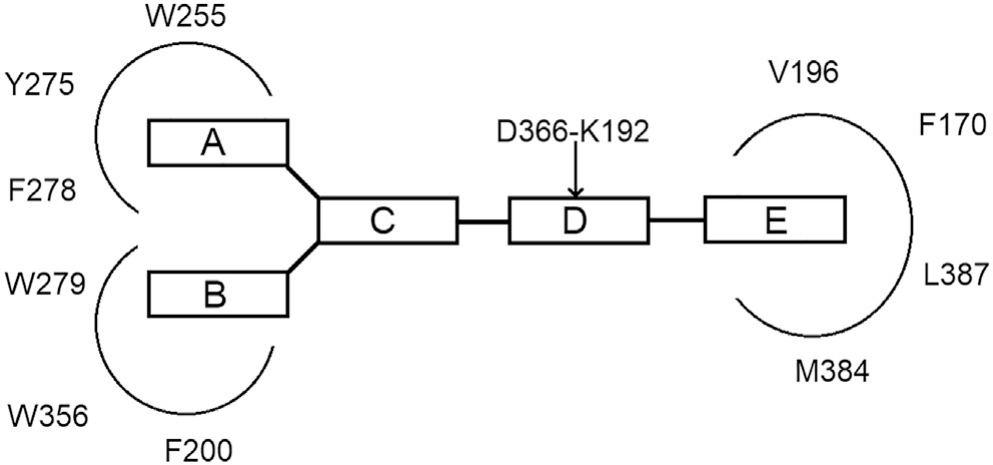
General CB_1_ Receptor Inverse Agonist Pharmacophore Model in Relation to the Putative CB_1_ Receptor Side Chain Residues in Receptor-Ligand Interactions Figure adapted from Lange and Kruse (2005).

An example of the success of this approach to advance experimental SAR work is provided by Lange and coworkers, who used computational methods to derive a general model of the five structural requirements of a CB_1_ inverse agonist pharmacophore (Figure 6): two aromatic moieties (A and B), a central unit (C), a hydrogen bond acceptor unit (D), and a cyclic lipophilic domain (E) (Lange and Kruse, 2005; De Azevedo and Russo, 2018).

However, there were clear limitations. Rhodopsin-based models lack the key N-terminal domain that shields the bound ligand from the solvent in CB_1_. To compensate, Reggio et al. used the human S1P1 receptor, with very high (62%–64%) sequence homology with the CB_1_ receptor in their TMs, for homology modeling (Hurst et al., 2013). They identified the two features of the S1P1 X-ray crystal that are particularly relevant for CB_1_ and CB_2_: an extracellular pocket that is closed off to the solvent and a gap between TM1 and TM2 that allows a ligand to pass from the lipid bilayer into the binding pocket. The eventual CB_1_ crystal structure determined by Liu in 2016 (Hua et al., 2016) proved very similar to this obtained homology model. The model was used to demonstrate that ligands such as **AEA** and **THC** access the CB_1_ binding site from the membrane by passing between residues F174^2.61^ and F177^2.64^, which facilitate transit by forming π−π interactions with the ligands (Jakowiecki and Filipek, 2016). This observation remains accurate.

Although the 2016 release of the first CB_1_ crystal structure increased the scope of modeling opportunities, homology modeling has continued to be an important approach to obtain accurate 3D structures in the absence of experimental structures, especially for the phytocannabinoids. In 2018, Liu and coworkers used a combination of sequence alignment and homology modeling of the S1P1 crystal structure (PDB: *3V2W*) (Hanson et al., 2012) to investigate the binding modes of rimonabant analogs as selective inverse agonists/antagonists for CB_1_ (Liu et al., 2018). Their CB_1_ model was validated by superimposing the model and the crystal structure (**PDB**: *5TGZ*). Docking analyses showed that the hydrophobic interactions between the ligands and the hydrophobic pockets of CB_1_ account for most of the binding affinity. The CB_1_ K192^3.28^ on TM3, known to be important for ligand binding, engaged in indirect, rather than direct, interactions with antagonists to keep the inactive-state CB_1_ stable through a salt bridge with D176^2.63^ on TM2. Another recent example is the high-throughput screen of over 3,000 CB_1_ antagonists by Liu et al., who modeled CB_1_ from the rhodopsin template. Multiple CB_1_ homology models were tested, and the best one was selected for use in virtual screening (Liu et al., 2014).

Homology modeling has been more important for CB_2_, and with the continuing lack of an agonist-bound form, remains essential. The CB_2_ model constructed from β1AR/β2AR/D3R has afforded better predictions for pre-screening compared with those built from the seven other GPCR crystal structures examined, namely, SMO (Wang et al., 2013), bovine rhodopsin (Palczewski et al., 2000), CXCR4 (Wu et al., 2010), M2MAR (Haga et al., 2012), D3R (Chien et al., 2010), β2AR (Cherezov et al., 2007), A2AAR (Lebon et al., 2011), H1R (Shimamura et al., 2011), S1P (Hanson et al., 2012), and β1AR (Warne et al., 2011). It is also suggested that sodium ions may reduce the binding affinity of endogenous agonists or its analogs (Feng et al., 2014). After the crystal structure of CB_1_ was reported in the literature in 2016, several studies used it as the basis for homology models of CB_2_. Makriyannis and coworkers developed a series of fluorescent ligands to better understand CB_2_ receptor expression and signaling to accelerate drug discovery (Mallipeddi et al., 2017). The release of the first CB_1_ crystal structure alignment (MUSTANG) of the **AM1336@CB**_**2**_ homology model showed an root-mean-square deviation of 2.10 Å and over 193 aligned residues with 47.7% sequence homology (Mallipeddi et al., 2017). **AM11542@CB**_**1**_ (**PDB**: *5XRA*) was also used for the generation of a homology model of a CB_2_-ligand complex for further computational studies (Dolles et al., 2018). As noted, this CB_1_ agonist provided a model of an antagonized CB_2_ system.

**Figure 7:**
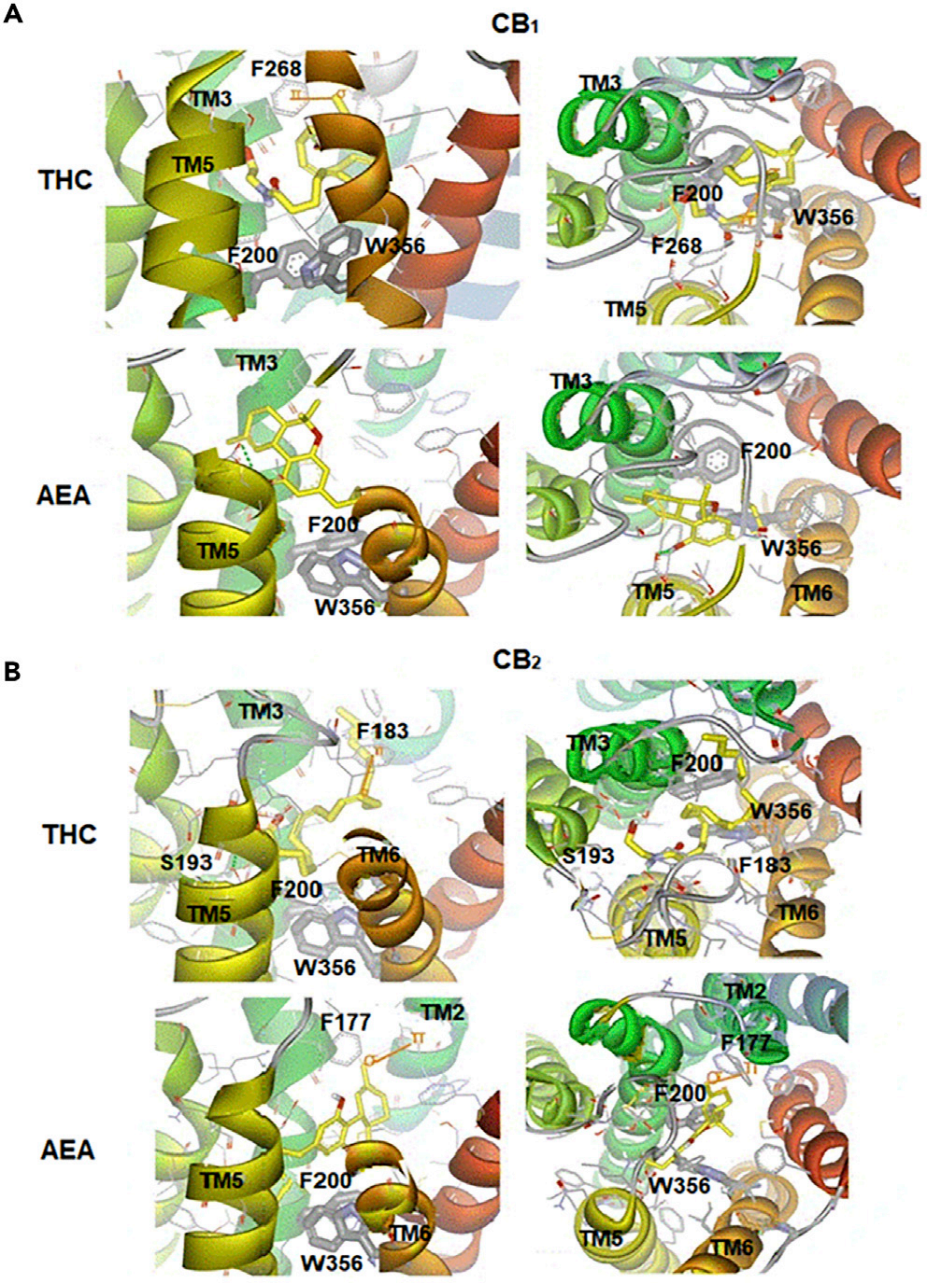
THC and AEA Bind to Both CB1 and CB2 Nonselective agonists THC and AEA bound to (A) CB_1_ and (B) CB_2_ in complexes characterized by the lowest energies; both top-down and side-on views are provided. Interactions involving π orbitals are shown as orange solid lines, and hydrogen bonds are shown as green dashed lines. Figure reproduced from Latek et al., (2011), with permission.

Latek et al. used structures of α2AR, β2AR, and β1AR as templates for homology modeling of CB_1_ and CB_2_ to bound **THC** and **AEA** in their crystal structures, combining induced docking of **THC@CB**_**1**_ and molecular dynamics simulations of the crystal structure of **THC@β2-adrenergic** receptor to provide insight (Latek et al., 2011). They revealed that the conformation of the centrally located W356^6.48^ and F200^3.36^ toggle switch differentiates between agonists and antagonists: nonselective agonists **THC** and **AEA** changed the positions of these two residues in both receptors relative to the empty receptor. **THC**s hydroxyl group formed a hydrogen bond with the δ-carboxylic acid E273^5.37^ in **THC@CB**_**1**_, sliding its alkyl tail slid into the hydrophobic channel between TMs 3, 6, and 7 close to the toggle switch (Figure 7A). In **THC@CB**_**2**_, **THC** forms the exact same hydrogen bond with E273^5.37^, but the alkyl chain sits in a slightly different position between TMs 3, **5**, and 6, but still leaving it close to the switch. The C5-6-fused bicycle of the ligand interacts with F177^2.64^ (σ-π interactions, Figure 7B) to lock-in this conformation. Endocannabinoid **AEA@CB**_**2**_ adopts a similar overall binding mode, with the nitrogen atom forming a hydrogen bond with S193^5.42^. The ligand's alkyl tail extends along the hydrophobic pocket defined by TM2 and TM3, interacting with phenylalanines F174^2.61^, F177^2.64^, and F189^3.25^ and the hydrophobic face of K192^3.28^ (Figure 7B).

Consequently, although experimentally validated structures are now available, homology modeling will remain an important approach for investigating interactions between ligands and the CB_2_ receptor and is expected to continue in this crucial role for years to come.

### Interactions between CB_1_/CB_2_ and the Phytocannabinoids: Case Studies

As we have no experimental structures of phytocannabinoids bound to CB_1_ or CB_2_, but do have them bound to other GPCRs, a combination of computational and experimental structural biology is required to make inferences about their binding and to determine the design of new ligands. The rest of the review is devoted to discussing the work done in this field to date for the most important cannabinoids.

### Δ^9^-Tetrahydrocannabinol (**THC**)

**THC** exhibits partial agonist activity at CB_1_ (K_*i*_ = 10 nM) and CB_2_ receptors (K_*i*_ = 24 nM) (Howlett et al., 2002). A detailed description of the downstream biological effects of **THC** binding to CB_1_ is beyond the scope of this review, but it downregulates secondary messenger molecule cAMP by inhibition of adenylate cyclase. This results in the observed psychotropic effects such as euphoria, relaxation, and anti-nociception (Hua et al., 2016). CB_1_ agonism by **THC** has broader pharmacological implications as it is used therapeutically as an analgesic, antiemetic, and anticonvulsant across the world. Being a partial rather than a full agonist of CB_1_, **THC** demonstrates lower cytotoxicity compared with synthetic cannabinoids like **FUB**, and consequently has a better safety profile.

**Figure 8:**
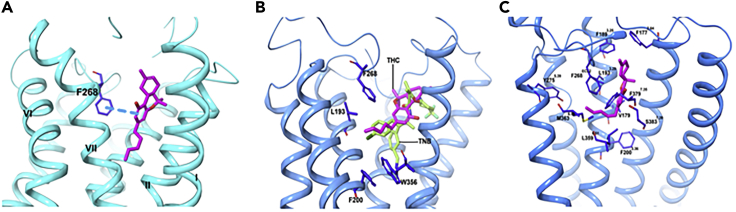
THC and TNB Adopt Different Comformations when Bound to CB1 (A–C) (A) Chemical structures and predicted binding poses of **THC** with inactive structure, (A) PBD: *5TGZ* and (B) PDB: *5U09* (**15**) (transparent magenta sticks), and (C) with active structure PDB: *5XRA*. **THC** (pink) and **TNB** (light green). Data used to prepare the figures were obtained from Hua et al., 2016 Figure 8A; Shao et al. (2016), Figure 8B; and Hua et al., 2017, Figure 8C.

When Hua et al. examined **THC@CB**_**1**_ using docking studies based on their CB_1_ crystal structure (**PDB:** *5TGZ*); (Hua et al., 2020) they predict that **THC** mainly interacts with ECL2, the N-terminal loop, and TM3, TM6, and TM7, but not with TM1 or TM2 (Figure 8A).The **THC** ring system resides between the N-terminal loop and ECL2, participating in π-π interactions with F268^ECL2^, whereas the carbon chains extend into the long hydrophobic pocket to interact with TM3, TM6, and TM7. In a second docking study of **THC@CB**_**1**_ based on the **TNB@CB**_**1**_ crystal structure discussed earlier, Shao and coworkers predicted that the tricyclic core of **THC** positions between TM1, TM2, and TM7 (as with **TBN**) (Shao et al., 2016), with the C_3_ alkyl chain overlapping with the chlorophenyl moiety of **TBN** and extending toward toggle residue W356^6.48^ (Figure 8B). Finally, C355^6.47^ on the external face of TM6 can form a covalent adduct with a **THC** analog that possesses a thiol at the end of the C_3_-pentyl chain (Picone et al., 2005). This obviously requires a rotation of TM6 during CB_1_ activation and consequent disruption of the packing around W356^6.48^; ligand binding is clearly dynamic. One caveat to these calculations, however, is that the inactive structure of CB_1_ used in these studies is not ideal for predicting high-affinity agonist interactions as an agonized binding site must access a different conformation in the presence of agonists to induce a conformational change to allow for G_i_ protein binding. In the third study by Hua et al., cited previously, the active crystal structure of CB_1_ was used (PDB: *5XRA*). The binding mode of **THC** was seen to resemble that of **AM11542@CB**_**1**_ (Figure 8C). The binding affinity of **THC** for CB_1_ results mainly from a combination of hydrophobic and aromatic interactions and excellent shape complementarity, rather than dipole-dipole or hydrogen bonding, with residues from ECL2 and TM3, TM5, TM6, and TM7. The tricyclic **THC** ring system forms π–π stacking with F268^ECL2^, F379^7.35^, F189^3.25^ and F177^2.64^, and the phenolic -OH at C_1_ forms a hydrogen bond with S383^7.3^. The alkyl chain of the **THC** extends into the long channel formed by TM3, TM5, and TM6, participating in hydrophobic interactions with L193^3.29^, V196^3.32^, Y275^5.39^, and L276^5.40^. **THC**, with a shorter alkyl chain (C_5_) compared with **AM11542**, shows a similar interaction. Daines and co-workers in 2018 looking for other docking sites on CB_1_ failed to identify any and reiterated the importance of residues 380–384 for binding (Sabatucci et al., 2018).

In 2019, Jung docked **THC** onto structures of both the inactive and the now available active conformations of CB_1_ (**PDB**: *5TGZ* and *5XRA*, respectively) and carried out molecular dynamic simulations to predict the binding poses of **THC** in the orthosteric ligand-binding site (Jung et al., 2018). **THC** docked well and adopted similar configurations in both conformations and was consistent with the homology modeling work discussed earlier. When **THC** was bound to the active conformation, it stably interacted with F200. The molecular mechanics Poisson-Boltzmann surface area (MM/PBSA) binding energies for the inactive and active conformations of CB_1_ with **THC** were −20.87 and −30.05 kcal/mol, respectively, supporting **THC**'s role as a partial agonist (Pertwee, 2008).

Tham et al. replicated the study, docking **THC** to the same crystal structures. In the agonist-bound receptor model *5XRA*, **THC** interacted with TM2, TM3, TM5, and TM7, whereas in the antagonist-bound model (*5TGZ*), **THC** showed weaker interactions with TM1, TM3, TM4, TM6, and TM7. The only interaction maintained in both conformations was between THC and TM3 T197^3.33^. Ligand affinity docking score values estimated by AutoDock for inactive *5XRA* and active *5TGZ* structures were −8.7 and −10.4 kcal.mol^−1^, respectively, in agreement with Jung.

Kumar et al. proposed that the lack of toggle switch interaction explains why **THC** has only partial CB_1_ agonist activity compared with the full agonist **AM series** (Krishna Kumar et al., 2019). Their docking calculations of **THC@CB**_**1**_ yield several poses where the terpenoid ring aligns well with that of the ligand in **AM11542@CB**_**1**_ (Hua et al., 2017); however, **THC**'s C3 alkyl chain was more flexible, able to both occupy the hydrophobic cavity, coincident with the *p*-fluorobenzyl group of full-agonist **FUB**, and to adopt a downward conformation where it points toward the toggle switch to activate the receptor. This seems to indicate that the conformational flexibility of **THC** in the orthosteric pocket likely modulates both its affinity for, and activating ability of, CB_1_, a characteristic that presumably makes it safer than the more rigid and potent “always-on” synthetic cannabinoids.

The computational study of the binding of THC to CB_2_ is more limited, but Vijayakumar and co-workers (Vijayakumar et al., 2019) determined the energetic docking scores of various with the receptor. They indicated that **THC** possesses superior docking scores (−5.379 kcal/mol) compared with known agonists such as **TNB**, **HU-308**, and **rimonabant**.

### Cannabidiol (**CBD**)

**CBD** is the second most abundant phytocannabinoid present in cannabis (Mechoulam et al., 2007) and accounts for up to 40% of dry mass in some cultivars. It is a partial agonist of the CB_2_ receptor, although it can bind to other, non-cannabinoid receptors, too. Preliminary clinical data suggest that **CBD** may ameliorate the symptoms of anxiety, cognitive and movement disorders, pain, and epileptic seizures. As of 2019, the specific mechanisms of action for its biological effects remain unclear. **CBD** has low affinity for the CB_1_ orthosteric site (McPartland et al., 2007), and is a CB_1_ antagonist (Thomas et al., 2007), but Laprairie et al. have reported that **CBD** might largely behave as a CB_1_ negative allosteric modulator (NAM) of **THC** and **2-AG** agonism (Laprairie et al., 2015).

In 2018, Sabatucci and colleagues (Sabatucci et al., 2018) performed an *in silico* docking study on the crystal structure of CB_1_ to find putative allosteric sites for **CBD**, and to examine its binding affinity. They identified three different sites based on a cluster analysis and calculated the binding energy (Table 3):

1. *Pocket 1*: in the transmembrane region between TM2 and TM4, ligand binding happens through the conserved residue W241 (TM4), hydrophobic residue F237 (TM4), and an electrostatic interaction with protonated H154 (TM2).
2. *Pocket 2*: between TM1, TM7, and intracellular helix 8.
3. *Pocket 3*: in the N-terminal region of CB_1_, partially overlapping the orthosteric site. It comprises the residue C107; however, interactions in this region were not completely characterized.
4. The orthosteric site: in the inactive conformation, the volume of the ligand-binding pocket is around 50% greater than in the active conformation.

**Table 2:** Summary of CB_1_ Agonist-Bound (*5XRA*) and Antagonist-Bound (*5TGZ*) Amino Acid Residues Interacting with Cannabinoid THC and CBD in AutoDock

TM	THC				CBD			
	*5XRA*		*5TGZ*		*5XRA*		*5TGZ*	
	CB1	CB2	CB1	CB2	CB1	CB2	CB1	CB2
1			M103^Nt^					S19^Nt^
						K23^Nt^		K23^Nt^
							M109^Nt^	
							Q115^1.31^	
							Q116^1.32^	
							I119^1.35^	
2		F87^2.57^		F87^2.57^				
		S90^2.60^						
						F91^2.61^		
	F170^2.57^							
	F174^2.61^							
	F177^2.64^				F177^2.64^			
							H178^2.65^	
						L182^ECL2^		L182^ECL2^
		F183^ECL2^				F183^ECL2^		F183^ECL2^
3		T114^3.33^		T114^3.33^				T144^3.33^
	L193^3.29^							
	T197^3.33^		T197^3.33^					
4			F268^4.77^		F268^4.77^			
5				W194^5.43^				
	W279^5.43^							
7				W258^6.48^				
					L359^6.51^			
			M363^6.55^					
Helix8		A282^7.36^				A282^7.36^		
				L289^7.43^				
	F379^7.34^				F379^7.34^			
	S383^7.39^		S383^7.39^

**Table 3:** Binding of THC, CBD, AEA, and 2-AG at the Various Potential Pockets on CB_1_

Modulators	Pocket 1	Pocket 2	Pocket 3	Orthosteric Site
	ΔG (kcal/mol)	ΔG (kcal/mol)	ΔG (kcal/mol)	ΔG (kcal/mol)
*THC*				−8.34
*CBD*	−7.75	–	−7,39	−8.15
*AEA*				−9.55
*2-AG*				−9.41

**Figure 9:**
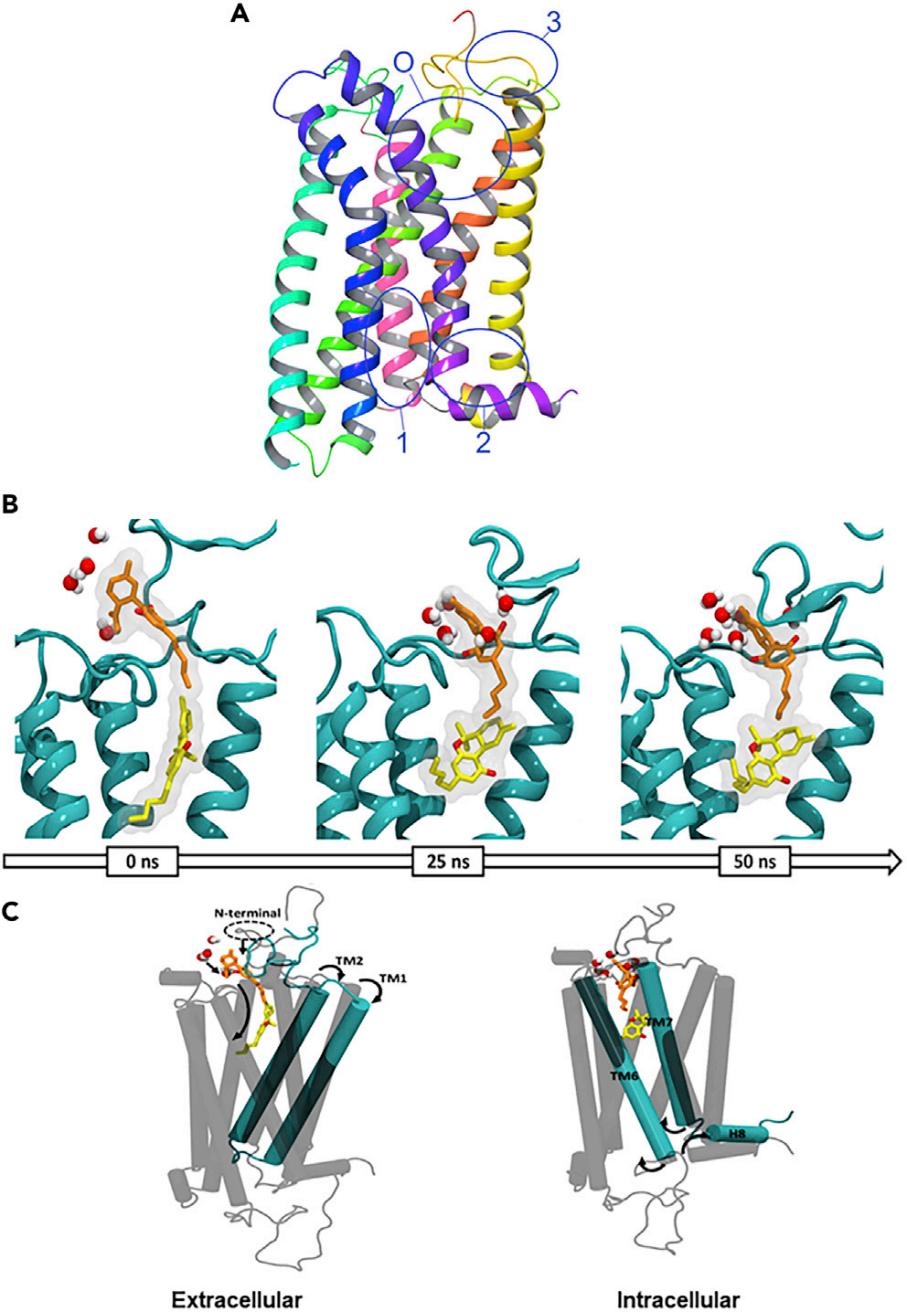
CBD and THC May Bind Simultaneously to CB1 (A–C) (A) Orthosteric site (O) and the three potential allosteric binding pockets in CB_1_ PDB: *5TGZ*. (B) Solvent rearrangement around **CBD** in the agonist-bound CB_1_. **CBD** (orange), **THC** (yellow), and water molecules within ≥3 Å in the active conformation of the CB1R at 0, 25, and 50 ns of simulation; (C) coordinated movement observed in the active conformation of CB_1_ during 50 ns of simulation. Data used to prepare (A) was obtained from Sabatucci et al., 2018; Figures 9B and 9C are reproduced from Chung et al. (2019) under Creative Commons Rights.

Examining **THC** as a control, they found, consistent with all the studies mentioned earlier, that **THC** binds only to the orthosteric site. No crystal structure of **CBD@CB**_**1**_ is available to answer this question, and it is possible that all three binding sites are in dynamic equilibrium (Figure 9A).

In their 2019 article discussed earlier using both the agonist- and antagonist-bound CB1 crystal structures, Tham et al. (2019) also studied potential **CBD@CB**_**1**_ interactions. **CBD** behaved as a NAM and occupied a ligand binding site in the antagonist-bound CB_1_ receptor model (*5TGZ*) that was separate from the orthosteric agonists tested. Table 2 summarizes the findings of Tham et al. **CBD** appears to have fluid affinity for both the allosteric (inactive state, R) and orthosteric (active state, R∗) sites at CB_1_ receptors, depending on whether another ligand is activating the receptor or not. Ligand affinity values estimated for the *5XRA-*CB_1_ and *5TGZ-*CB_1_ receptor models were −7.1 and −9.7 kcal.mol^−1^, respectively. On the basis of these data, it is evident that **CBD** has a high affinity for an allosteric site in the outer vestibule of the antagonist-bound CB_1_ receptor. To analyze the effect that **CBD** binding has on the activated receptor, a 50-ns molecular dynamics simulation was performed by Chung and coworkers on the **CBD@(THC@CB**_**1**_) complex (Chung et al., 2019). **CBD** binding leads to coordinated opening of both the cytoplasmic and extracellular pockets, allowing improved access for ligands in the binding site while closing off the G_i_ binding site. Hydration of **CBD**'s exposed terpenoid ring leads to folding of the membrane proximal region, which both promotes partial entry of **CBD** into the orthosteric binding site in the presence of **THC,** which adopts an L-shape conformation to accommodate the new ligand (Figure 9B). To expand the orthosteric site to accommodate two small molecules, TM1 and TM2 pincer apart on the extracellular side, whereas TM6 and TM7 do the same on the intracellular side (Figure 9C).

The N-terminal loop formed by the C98-C107 disulfide bond then closes over **CBD** as it enters deeper into the binding site. This cap is anchored by a semi-organized water blanket that seals the pocket behind **CBD**. From here, **CBD** tightens its affinity for the site by interacting with ECL2 and the N-terminal loop, forming hydrogen bonds with I267 and well-organized capping water molecules. Despite **CBD**'s ability to bind to multiple sites of CB_1_ in the presence of **THC**, this work makes a strong argument that the preferred binding site is located at the entry to the orthosteric binding site: **CBD** follows **THC** into the receptor, turning the GPCR off just as the THC turns it on.

Docking studies conducted on a **CBD@CB**_**2**_ homology model built on **AM11542@CB**_**1**_ and **AM6538@CB**_**1**_ display partial agonism and orthosteric site binding through key interactions with residues K23^Nt^, F91^2.61^, L182^ECL2^, FECL2^183^, and A282^7.36^ in the *5XRA* model. The two different models provide slightly different conformations, with estimated binding energy being −8.9 and −9.4 kcal/mol, respectively (Table 4) (Tham et al., 2019). This is different from the preferences of **THC@CB**_**2**_ (analyzed by homology modeling of both **AM11542@CB**_**1**_ and **AM6538@CB**_**1**_, both producing similar results), which interacts with only two of the same key residues. Ligand affinity of **THC** estimated for homology modeling of CB2 with the *5XRA* and *5TGZ* receptor models was −9.4 and −8.9 kcal.mol^−1^, respectively.

**Table 4:** Summary of CB_2_ Agonist-Bound (*5XRA*) and Antagonist-Bound (*5TGZ*) Amino Acid Residues Interacting with Cannabinoid THC and CBD

TM	THC		CBD	
	CB_2_		CB_2_	
	*5XRA*	*5TGZ*	*5XRA*	*5TGZ*
1				S19^Nt^
			K23^Nt^	K23^Nt^
2	F87^2.57^	F87^2.57^		
	S90^2.60^			
			F91^2.61^	
			L182^ECL2^	L182^ECL2^
	FECL2^183^		FECL2^183^	FECL2^183^
3	T114^3.33^	T114^3.33^		T114^3.33^
5		W194^5.43^		
6		W258^6.48^		
7	A282^7.36^		A282^7.36^	
		L289^7.43^		

There are isolated reports of the modeling of various other phytocannabinoids such as **CBG** (El-Darawy et al., 1972) and **CBN** (El-Darawy et al., 1972). However, the work on the molecular modeling of these systems remains very preliminary, although it will likely greatly expand in the coming years. We anticipate that an update on this review will likely focus more on these minor cannabinoids as much of the pharmacological activity ascribed to cannabis might lie with these minor compounds rather than exclusively with the two major cannabinoids. However, one of the “major” minor cannabinoids, **THCV,** has drawn some attention (Gill, 1971).

### Δ^9^-Tetrahydrocannabivarin (**THCV**)

**THCV** is structurally similar to **THC**, with the only difference being two fewer carbons in the carbon tail: both molecules share similar traits, binding affinities, and metabolic derivatives. However, unlike **THC**, **THCV** has proposed anti-obesity activity. **THCV**'s activity is complicated: it is implicated as a cannabinoid receptor antagonist by competitively inhibiting **THC@CB**_**1**_ formation, although other evidence implies that **THCV** may also be an indirect agonist (Englund et al., 2015).

In 2018, as part of their extensive analysis, Jung and coworkers docked **THCV** to both the inactive and active conformations of CB_1_ as extracted from the crystal structures of *5TGZ* and *5XRA*, respectively (Jung et al., 2018). **THCV** docked well to both conformations, exhibiting similar binding poses in the orthosteric ligand-binding site, with binding affinities to the inactive and active conformations (MM/PBSA) being −21.02 and −28.03 kcal⋅mol^−1^ respectively. The only difference between **THCV** and **THC** was that the pentyl side chain of **THC** protrudes into the sub-pocket of the binding site, which does not occur during **THCV** binding (Jung et al., 2018). Both **THC** and **THCV** interacted more favorably with the active conformation than the inactive conformation, although **THCV** showed lower affinity for the active conformation than **THC**.

**Figure 10:**
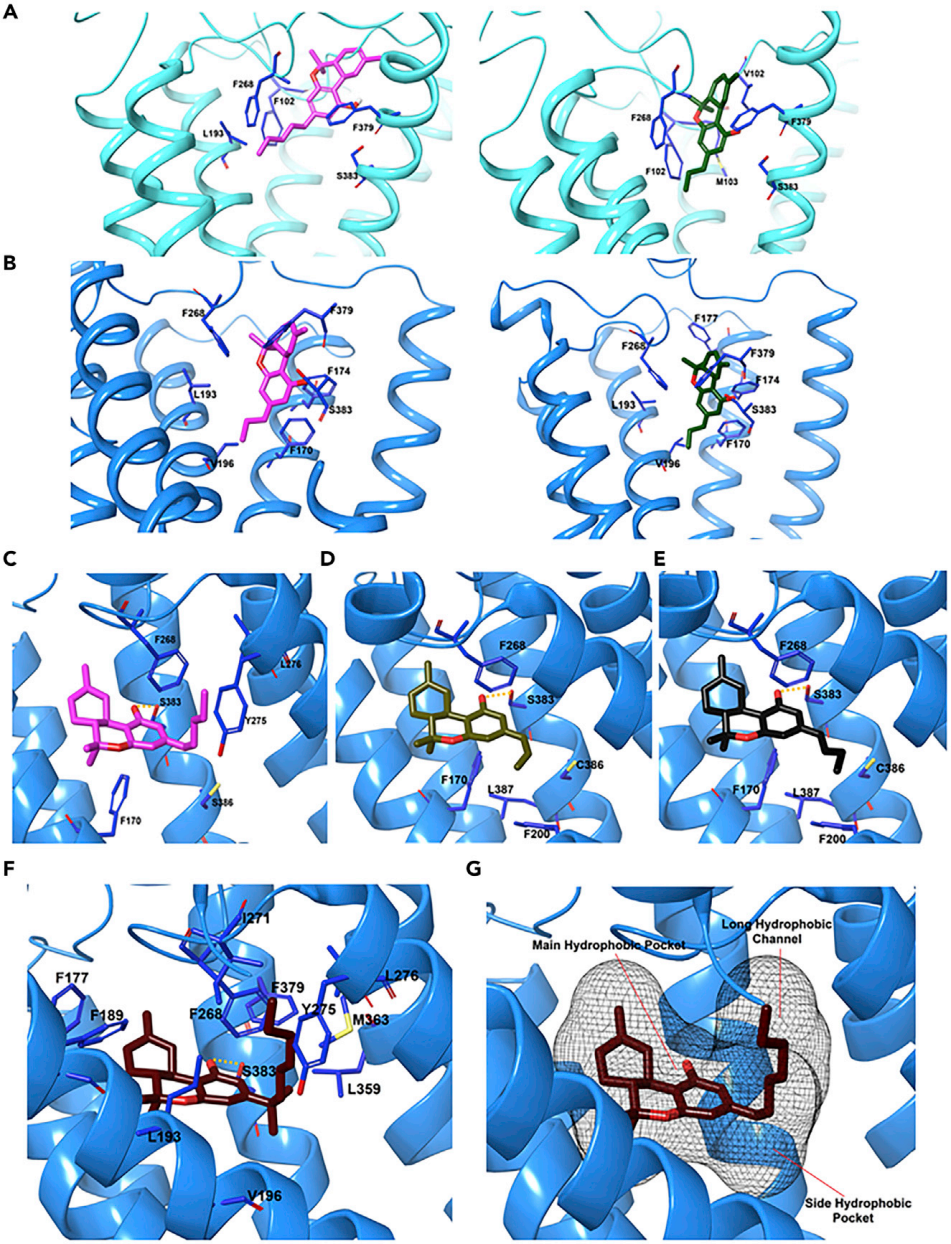
Binding Poses of CB_1_ Ligands after 1-μs Molecular Dynamics Simulations (A–G) THC and THCV were bound to the (A) inactive and (B) active conformations of CB_1_ receptor. Docking pose of (C) **THC,** (D) **THCV**, (E) **THCB**, and (F and G) **THCP** in complex with CB_1_ receptor (PDB ID: *5XRA*). Data used to prepare Figures 10A and 10B were obtained from Jung et al. (2018), and for Figures 10C–10G from Citti et al. (2019a, 2019b).

In the inactive conformation of **THCV@CB**_**1**_, **THCV** interacts with the same three residues as **THC**: F102, F379, and S383, but it also interacts with three other residues: M103, I105, and F268. These additional hydrophobic attractions are sufficiently weak that **THC** retains stronger affinity for the orthosteric binding site (Figures 10A and 10B). In the active conformation, both **THC** and **THCV** interact with five common residues: F170, L193, V196, F268, and F379, whereas **THCV** also binds to F177. Both ligands are strong binders to the orthosteric site, although **THC** does show more potent activity (Jung et al., 2018).

### Tetrahydrocannabutol (**THCB**) and Cannabidibutol

One of the essential parameters that can affect the biological activity of **THC**-like cannabinoids is the length of the C_*3*_ alkyl chain. A minimum of three carbons are necessary for receptor binding; activity then increases with increasing number of carbons up to a maximum of eight carbons; for n > 8, activity begins to decrease again (Bow and Rimoldi, 2016). Cannazza and colleagues have recently reported two new phytocannabinoids, tetrahydrocannabutol (**THCB**) and cannabidibutol, both with linear alkyl side chains containing four carbon atoms (Citti et al., 2019a; Linciano et al., 2020). **THCB** has thrice the affinity of **THC** for CB_1_, with unchanged affinity for CB_2_ (Bow and Rimoldi, 2016). Docking simulations of **THCB@** CB_1_ found binding poses similar to those exhibited by **THC** and **THCV**. The main difference between the three ligands was observed in the position of the aliphatic side chain: the butyl chain of **THCB** does not extend down the TM3-TM5-TM6 tunnel like the pentyl chain of **THC** (Figure 10C); instead, like the propyl chain of **THCV** it binds to the small hydrophobic subpocket located near the entrance of the tunnel (Figure 10D). This leads to **THCB**'s optimal hydrophobic interaction with F170, F200, and L387, accounting for its exceptional affinity for the CB1 receptor (Figure 10E).

### Tetrahydrocannabiphorol (**THCP**) and Cannabidiphorol (**CBDP**)

In late 2019, Cannazza and coworkers reported the isolation of two additional phytocannabinoids: tetrahydrocannabiphorol (**THCP**) and cannabidiphorol, both with C_*7*_ linear alkyl side chains (Citti et al., 2019b). **THCP** has exceptional affinity to both CB_1_ and CB_2_, with a K_*i*_ of 1.2 and 6.2 nM, respectively, 33-fold the affinity of **THC** for CB_1_ and 13-fold higher than **THCB**. **THCP** binds to the active conformation of CB_1_ in an L-shaped pose, approximating that of **THC** in the presence of **CBD** (Chung et al., 2019), with the tetrahydro-6H-benzo[c]chromene ring system occupying the hydrophobic orthosteric pocket. The resorcinyl moiety participates in two edge-to-face π-π interactions with F170 and F268, and the C_*1*_ hydroxyl group hydrogen bonds with S383 (Figure 10F). Interestingly, the heptyl chain at C_*3*_ extended into a long hydrophobic tunnel formed by L193, V196, Y275, I271, L276, W279, L359, F379, and M363, formed by TM3, TM5, and TM6, along its entire length, maximizing the hydrophobic interactions with the residues along the side of the channel (Figures 10F and 10G). In contrast, the tunnel is only partially occupied by the shorter pentyl chain of **THC**, helping to account for **THCP**'s far higher affinity. This orientation is also different than the pose predicted for the shorter alkyl chain homologs, **THCV** and **THCB,** where the chain sits in the side hydrophobic pocket instead. In a murine model, **THCP** showed promising pharmacological activities associated with CB_1_ agonists including reduced motility, analgesia, catalepsy, and decreased rectal temperature.

### Endocannabinoids (**AEA** and **2-AG**)

Finally, the binding interactions between the endogenous cannabinoids have received some attention (Stella et al., 1997; Maccarrone, 2019). The first endogenous cannabinoid, **AEA**, was isolated from porcine brain (Devane et al., 1992) and was shown to bind to the CB_1_ receptor with high affinity. **2-AG**, a second endogenous cannabinoid, was isolated from intestinal tissue of mice (Mechoulam et al., 1995). **2-AG** has been found present in the brain at concentrations 170 times greater than **AEA**. Furthermore, **2-AG** acts as a full agonist, producing the characteristic effects associated with cannabinoid agonists (Stella et al., 1997).

**Figure 11:**
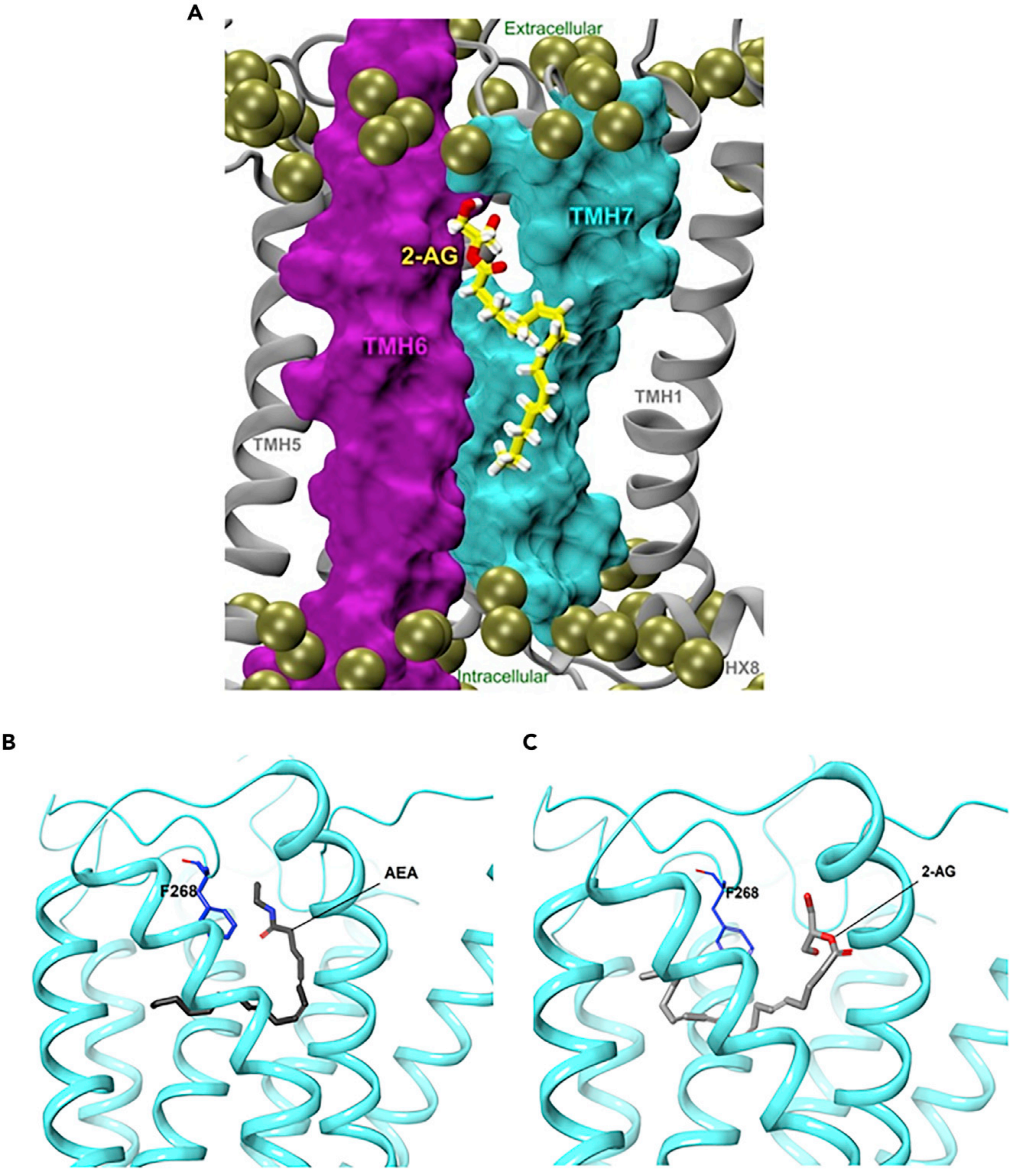
Endogenous Cannabinoids Interact with the CB2 Receptor (A) Molecular dynamics simulations of 2-AG binding to the membrane-embedded CB_2_ receptor. Predicted binding poses of (B) AEA and (C) 2-AG. Figure 11A is reproduced from Hurst et al. (2010) with permission; data used to generate Figures 11B and 11C were obtained from Hua et al.

**AEA** has low CB_2_ affinity, whereas **2-AG** binds strongly to CB_2_ (Mechoulam et al., 1995; Sugiura et al., 1995). Microsecond timescale molecular dynamics simulations of the interaction of **2-AG** with CB_2_ receptor in a palmitoyl-oleoylphosphatidylcholine lipid bilayer have provided insight into the mechanism of binding. **2-AG** partitions out of bulk lipid at the TM6/7 interface, and then enters the CB_2_ receptor binding pocket by passing between TM6/7 (Figure 11A), similar to the phytochemicals. The entrance of the 2-AG head group into the CB_2_ binding pocket is sufficient to trigger breaking of the intracellular TM3/6 ionic lock and cause the movement of the TMH intracellular end away from TM3 (Hurst et al., 2010). Both **AEA** and **2-AG** are predicted to adopt a C-shaped conformation and occupy a similar position in the inactive structure as **THC** (PDB; *5TGZ*; Figures 11B and 11C), but their longer shape means that their hydrophilic heads rest between the N-terminal loop and ECL2 and their long tails stretch deeper into the hydrophobic tunnel, like **THCP** (Hua et al., 2016).

### Conclusion and Perspectives

As the residual stigma associated with cannabis use is reduced by spreading legalization, and as additional clinical reports on phytocannabinoid activity begin to proliferate, it is increasingly essential to understand the molecular basis of activity. This is clearly not restricted to the CB_1_ and CB_2_ alone, but preliminary work has focused on these obvious candidates. The structural biology work outlined in this article, both experimental and computational, has largely supported the hypotheses laid out in the point mutation studies, and progress has been very rapid despite the fact that the crystal structures of any of the cannabinoid receptors are no more than 4 years old at the time of writing. Far from being associated with only psychoactive effects, the cannabinoid binding sites have been found to be the major mediators of important physiological functions such as analgesia, hunger, emesis, and immunochemical responses. Many of these effects likely arise from a complicated interplay of agonism and antagonism of CB_1_, CB_2_, and other related GPCRs; the activation profiles of the major cannabinoids are clearly very poorly understood. However, a far more fruitful area of future development will likely involve detailed studies on the minor phytocannabinoids (>150 at time of writing), some of which have activity orders of magnitude higher than that of **THC** and **CBD**.

The field is expected to advance rapidly in the coming months and years as additional cryo-EM and X-ray structures are solved; it is likely a matter of time before structures in the presence of key phytocannabinoids are available. The dynamism of these receptors, their interconversion between agonized and antagonized states, and a clear determination of the mechanism of action of partial agonists might be clarified by the complementary application of NMR-solution-phase structural analysis, biophysics techniques, and computational modeling. Much of the fundamental work has been done, but greater clarity regarding the relationship between structure and function remains a tempting challenge. In this review we seek to demonstrate that computational and experimental approaches work exceedingly well together and note with optimism that combined studies are becoming ever more common; we hope that this continues to be the case. This is especially important as the role of the cannabinoids in physiology likely extends far beyond the cannabinoid receptors, and we expect that future studies will also report on their interactions with other GPCRs and related proteins as we better understand their fascinating pharmacology.

A better understanding of these interactions will improve our understanding of neuropsychopharmacology and not only will help guide the development of new more selective drugs, and new cannabis cultivars with specific minor cannabinoid profiles to better realize the therapeutic potential of cannabis, but also will provide greater insight into the specific binding modes and interdependency of the GPCRs. We hope an update on this review will provide a much broader scope and far greater insight into these other drugs.

## Methods

All methods can be found in the accompanying Transparent Methods supplemental file.

## References

[bib1] Baker D., Pryce G., Giovannoni G., Thompson A.J. (2003). The therapeutic potential of cannabis. Lancet Neurol..

[bib2] Bouaboula M., Poinot-Chazel C., Marchand J., Canat X., Bourrié B., Rinaldi-Carmona M., Calandra B., Le Fur G., Casellas P. (1996). Signaling pathway associated with stimulation of CB_2_ peripheral cannabinoid receptor. Eur. J. Biochem..

[bib3] Bow E.W., Rimoldi J.M. (2016). The structure–function relationships of classical cannabinoids: CB_1_/CB_2_ modulation. Perspect. Medicin. Chem..

[bib4] Cherezov V., Rosenbaum D.M., Hanson M.A., Rasmussen S.G.F., Thian F.S., Kobilka T.S., Choi H.-J., Kuhn P., Weis W.I., Kobilka B.K. (2007). High-Resolution crystal structure of an engineered human β_2_-adrenergic G protein–coupled receptor. Science.

[bib5] Chien E.Y.T., Liu W., Zhao Q., Katritch V., Won Han G., Hanson M.A., Shi L., Newman A.H., Javitch J.A., Cherezov V. (2010). Structure of the human dopamine D3 receptor in complex with a D2/D3 selective antagonist. Science.

[bib6] Chung H., Fierro A., Pessoa-Mahana C.D. (2019). Cannabidiol binding and negative allosteric modulation at the cannabinoid type 1 receptor in the presence of Δ9-tetrahydrocannabinol: an *in Silico* study. PLoS One.

[bib7] Citti C., Linciano P., Forni F., Vandelli M.A., Gigli G., Laganà A., Cannazza G. (2019). Analysis of impurities of cannabidiol from hemp. Isolation, characterization and synthesis of cannabidibutol, the novel cannabidiol butyl analog. J. Pharmaceut. Biomed..

[bib8] Citti C., Linciano P., Russo F., Luongo L., Iannotta M., Maione S., Laganà A., Capriotti A.L., Forni F., Vandelli M.A. (2019). A novel phytocannabinoid isolated from *Cannabis sativa* L. with an in vivo cannabimimetic activity higher than Δ9-tetrahydrocannabinol: Δ9-Tetrahydrocannabiphorol. Sci. Rep..

[bib9] De Azevedo W., Russo S. (2018). Advances in the understanding of the cannabinoid receptor 1 - focusing on the inverse agonists interactions. Curr. Med. Chem..

[bib10] Devane W.A., Hanus L., Breuer A., Pertwee R.G., Stevenson L.A., Griffin G., Gibson D., Mandelbaum A., Etinger A., Mechoulam R. (1992). Isolation and structure of a brain constituent that binds to the cannabinoid receptor. Science.

[bib11] Dolles D., Hoffmann M., Gunesch S., Marinelli O., Möller J., Santoni G., Chatonnet A., Lohse M.J., Wittmann H.-J., Strasser A. (2018). Structure–activity relationships and computational investigations into the development of potent and balanced dual-acting butyrylcholinesterase inhibitors and human cannabinoid receptor 2 ligands with pro-cognitive *in vivo* profiles. J. Med. Chem..

[bib12] Durdagi S., Papadopoulos M.G., Zoumpoulakis P.G., Koukoulitsa C., Mavromoustakos T. (2010). A computational study on cannabinoid receptors and potent bioactive cannabinoid ligands: homology modeling, docking, de novo drug design and molecular dynamics analysis. Mol. Divers..

[bib13] El-Darawy Z.I., Roushdy M.I., Rizk A.M., Hammouda F.M., Mobarak Z.M. (1972). Studies on Hashish I. Isolation & identification of cannabinols and effect of certain factors. Qual. Plant Mater. Veg..

[bib14] Englund A., Atakan Z., Kralj A., Tunstall N., Murray R., Morrison P. (2015). The effect of five day dosing with THCV on THC-induced cognitive, psychological and physiological effects in healthy male human volunteers: a placebo-controlled, double-blind, crossover pilot trial. J. Psychopharmacol..

[bib15] Fahrenholtz K.E., Lurie M., Kierstead R.W. (1966). Total synthesis of *dl*-Δ^9^-Tetrahydrocannabinol and of *dl*-Δ^8^-tetrahydrocannabinol, racemates of active constituents of marihuana. J. Am. Chem. Soc..

[bib16] Feng Z., Alqarni M.H., Yang P., Tong Q., Chowdhury A., Wang L., Xie X.Q. (2014). Modeling, molecular dynamics simulation, and mutation validation for structure of cannabinoid receptor 2 based on known crystal structures of GPCRs. J. Chem. Inf. Model..

[bib17] Gill E.W. (1971). Propyl homologue of tetrahydrocannabinol: its isolation from Cannabis, properties, and synthesis. J. Chem. Soc. C.

[bib18] Haga K., Kruse A.C., Asada H., Yurugi-Kobayashi T., Shiroishi M., Zhang C., Weis W.I., Okada T., Kobilka B.K., Haga T. (2012). Structure of the human M_2_ muscarinic acetylcholine receptor bound to an antagonist. Nature.

[bib19] Hanson M.A., Roth C.B., Jo E., Griffith M.T., Scott F.L., Reinhart G., Desale H., Clemons B., Cahalan S.M., Schuerer S.C. (2012). Crystal structure of a lipid G protein–coupled receptor. Science.

[bib20] Hauser A.S., Attwood M.M., Rask-Andersen M., Schiöth H.B., Gloriam D.E. (2017). Trends in GPCR drug discovery: new agents, targets and indications. Nat. Rev. Drug Discov..

[bib21] Howlett A.C., Barth F., Bonner T.I., Cabral G., Casellas P., Devane W.A., Felder C.C., Herkenham M., Mackie K., Martin B.R. (2002). International union of pharmacology. XXVII. Classification of cannabinoid receptors. Pharmacol. Rev..

[bib22] Hryhorowicz S., Kaczmarek-Rys M., Andrzejewska A., Staszak K., Hryhorowicz M., Korcz A., Slomski R. (2019). Allosteric modulation of cannabinoid receptor 1- current challenges and future opportunities. Int. J. Mol. Sci..

[bib23] Hua T., Li X., Wu L., Iliopoulos-Tsoutsouvas C., Wang Y., Wu M., Shen L., Johnston C.A., Nikas S.P., Song F. (2020). Activation and signaling mechanism revealed by cannabinoid receptor-G_i_ complex structures. Cell.

[bib24] Hua T., Vemuri K., Nikas S.P., Laprairie R.B., Wu Y., Qu L., Pu M., Korde A., Jiang S., Ho J.H. (2017). Crystal structures of agonist-bound human cannabinoid receptor CB_1_. Nature.

[bib25] Hua T., Vemuri K., Pu M., Qu L., Han G.W., Wu Y., Zhao S., Shui W., Li S., Korde A. (2016). Crystal structure of the human cannabinoid receptor CB_1_. Cell.

[bib26] Huffman J.W., Yu S., Showalter V., Abood M.E., Wiley J.L., Compton D.R., Martin B.R., Bramblett R.D., Reggio P.H. (1996). Synthesis and pharmacology of a very potent cannabinoid lacking a phenolic hydroxyl with high affinity for the CB_2_ receptor. J. Med. Chem..

[bib27] Hurst D.P., Grossfield A., Lynch D.L., Feller S., Romo T.D., Gawrisch K., Pitman M.C., Reggio P.H. (2010). A lipid pathway for ligand binding is necessary for a cannabinoid G protein-coupled receptor. J. Biol. Chem..

[bib28] Hurst D.P., Schmeisser M., Reggio P.H. (2013). Endogenous lipid activated G protein-coupled receptors: emerging structural features from crystallography and molecular dynamics simulations. Chem. Phys. Lipids.

[bib29] Jakowiecki J., Filipek S. (2016). Hydrophobic ligand entry and exit pathways of the CB_1_ cannabinoid receptor. J. Chem. Inf. Model..

[bib30] Jensen A.D., Guarnieri F., Rasmussen S.G., Asmar F., Ballesteros J.A., Gether U. (2001). Agonist-induced conformational changes at the cytoplasmic side of transmembrane segment 6 in the β2 adrenergic receptor mapped by site-selective fluorescent labeling. J. Biol. Chem..

[bib31] Jung S.W., Cho A.E., Yu W. (2018). Exploring the ligand efficacy of cannabinoid receptor 1 (CB_1_) using molecular dynamics simulations. Sci. Rep..

[bib32] Krebs A., Villa C., Edwards P.C., Schertler G.F. (1998). Characterisation of an improved two-dimensional p22121 crystal from bovine rhodopsin. J. Mol. Biol..

[bib33] Krishna Kumar K., Shalev-Benami M., Robertson M.J., Hu H., Banister S.D., Hollingsworth S.A., Latorraca N.R., Kato H.E., Hilger D., Maeda S. (2019). Structure of a signaling cannabinoid receptor 1-G protein complex. Cell.

[bib34] Lange J.H., Kruse C.G. (2005). Medicinal chemistry strategies to CB_1_ cannabinoid receptor antagonists. Drug Discov. Today.

[bib35] Laprairie R.B., Bagher A.M., Kelly M.E., Denovan-Wright E.M. (2015). Cannabidiol is a negative allosteric modulator of the cannabinoid CB_1_ receptor. Br. J. Pharmacol..

[bib36] Latek D., Kolinski M., Ghoshdastider U., Debinski A., Bombolewski R., Plazinska A., Jozwiak K., Filipek S. (2011). Modeling of ligand binding to G protein coupled receptors: cannabinoid CB_1_, CB_2_ and adrenergic β2AR. J. Mol. Model..

[bib37] Latorraca N.R., Venkatakrishnan A.J., Dror R.O. (2017). GPCR dynamics: structures in motion. Chem. Rev..

[bib38] Lebon G., Warne T., Edwards P.C., Bennett K., Langmead C.J., Leslie A.G.W., Tate C.G. (2011). Agonist-bound adenosine A2A receptor structures reveal common features of GPCR activation. Nature.

[bib39] Li X., Hua T., Vemuri K., Ho J.-H., Wu Y., Wu L., Popov P., Benchama O., Zvonok N., Locke K.A. (2019). Crystal structure of the human cannabinoid receptor CB_2_. Cell.

[bib40] Lin S.W., Sakmar T.P. (1996). Specific tryptophan UV-absorbance changes are probes of the transition of rhodopsin to its active state. Biochemistry.

[bib41] Linciano P., Citti C., Luongo L., Belardo C., Maione S., Vandelli M.A., Forni F., Gigli G., Laganà A., Montone C.M. (2020). Isolation of a high-affinity cannabinoid for the human CB_1_ receptor from a medicinal *Cannabis sativa* variety: Δ^9^-Tetrahydrocannabutol, the butyl homologue of Δ^9^-Tetrahydrocannabinol. J. Nat. Prod..

[bib42] Liu C., Yuan C., Wu P., Zhu C., Fang H., Wang L., Fu W. (2018). Computational investigation on the binding modes of Rimonabant analogs with CB_1_ and CB_2_. Chem. Biol. Drug Des..

[bib43] Liu H., Patel R.Y., Doerksen R.J. (2014). Structure of the cannabinoid receptor 1: homology modeling of its inactive state and enrichment study based on CB_1_ antagonist docking. MedChemComm.

[bib44] Maccarrone M. (2019). Missing pieces to the endocannabinoid puzzle. Trends Mol. Med..

[bib45] Maccarrone M., Battista N., Centonze D. (2007). The endocannabinoid pathway in Huntington's disease: a comparison with other neurodegenerative diseases. Prog. Neurobiol..

[bib46] Mackie K. (2008). Cannabinoid receptors: where they are and what they do. J. Neuroendocrinol.

[bib47] Mahmoudian M. (1997). The cannabinoid receptor: computer-aided molecular modeling and docking of ligand. J. Mol. Graph. Model..

[bib48] Mallipeddi S., Kreimer S., Zvonok N., Vemuri K., Karger B.L., Ivanov A.R., Makriyannis A. (2017). Binding site characterization of AM1336, a novel covalent inverse agonist at human cannabinoid 2 receptor, using mass spectrometric analysis. J. Proteome Res..

[bib49] McAllister S.D., Hurst D.P., Barnett-Norris J., Lynch D., Reggio P.H., Abood M.E. (2004). Structural mimicry in class A G protein-coupled receptor rotamer toggle switches: the importance of the F3.36(201)/W6.48(357) interaction in cannabinoid CB_1_ receptor activation. J. Biol. Chem..

[bib50] McPartland J.M., Glass M., Pertwee R.G. (2007). Meta-analysis of cannabinoid ligand binding affinity and receptor distribution: interspecies differences. Br. J. Pharmacol..

[bib51] Mechoulam R., Ben-Shabat S., Hanus L., Ligumsky M., Kaminski N.E., Schatz A.R., Gopher A., Almog S., Martin B.R., Compton D.R. (1995). Identification of an endogenous 2-monoglyceride, present in canine gut, that binds to cannabinoid receptors. Biochem. Pharmacol..

[bib52] Mechoulam R., Peters M., Murillo-Rodriguez E., Hanus L.O. (2007). Cannabidiol -- recent advances. Chem. Biodivers..

[bib53] Montero C., Campillo N.E., Goya P., Paez J.A. (2005). Homology models of the cannabinoid CB_1_ and CB_2_ receptors. A docking analysis study. Eur. J. Med. Chem..

[bib54] Munro S., Thomas K.L., Abu-Shaar M. (1993). Molecular characterization of a peripheral receptor for cannabinoids. Nature.

[bib55] Nakanishi J., Takarada T., Yunoki S., Kikuchi Y., Maeda M. (2006). FRET-based monitoring of conformational change of the β2 adrenergic receptor in living cells. Biochem. Biophys. Res. Commun..

[bib56] Ogawa G., Tius M.A., Zhou H., Nikas S.P., Halikhedkar A., Mallipeddi S., Makriyannis A. (2015). 3′-Functionalized adamantyl cannabinoid receptor probes. J. Med. Chem..

[bib57] Palczewski K., Kumasaka T., Hori T., Behnke C.A., Motoshima H., Fox B.A., Trong I.L., Teller D.C., Okada T., Stenkamp R.E. (2000). Crystal structure of Rhodopsin: a G protein-coupled receptor. Science.

[bib58] Pertwee R.G. (2006). Cannabinoid pharmacology: the first 66 years. Br. J. Pharmacol..

[bib59] Pertwee R.G. (2008). The diverse CB_1_ and CB_2_ receptor pharmacology of three plant cannabinoids: Δ9-tetrahydrocannabinol, cannabidiol and Δ9-tetrahydrocannabivarin. Br. J. Pharmacol..

[bib60] Picone R.P., Khanolkar A.D., Xu W., Ayotte L.A., Thakur G.A., Hurst D.P., Abood M.E., Reggio P.H., Fournier D.J., Makriyannis A. (2005). (-)-7'-Isothiocyanato-11-hydroxy-1',1'-dimethylheptylhexahydrocannabinol (AM841), a high-affinity electrophilic ligand, interacts covalently with a cysteine in helix six and activates the CB_1_ cannabinoid receptor. Mol. Pharmacol..

[bib61] Reekie T.A., Scott M.P., Kassiou M. (2017). The evolving science of phytocannabinoids. Nat. Rev. Chem..

[bib62] Reggio P.H. (2010). Endocannabinoid binding to the cannabinoid receptors: what is known and what remains unknown. Curr. Med. Chem..

[bib63] Rinaldi-Carmona M., Barth F., Millan J., Derocq J.-M., Casellas P., Congy C., Oustric D., Sarran M., Bouaboula M., Calandra B. (1998). SR 144528, the first potent and selective antagonist of the CB_2_ cannabinoid receptor. J. Pharmacol. Exp. Ther..

[bib64] Rosenqvist E., Ottersen T. (1975). The crystal and molecular structure of Δ^9^-tetrahydrocannabinolic acid B. Acta Chem. Scand. B.

[bib65] Sabatucci A., Tortolani D., Dainese E., Maccarrone M. (2018). *In silico* mapping of allosteric ligand binding sites in type-1 cannabinoid receptor. Biotechnol. Appl. Biochem..

[bib66] Saroz Y., Kho D.T., Glass M., Graham E.S., Grimsey N.L. (2019). Cannabinoid receptor 2 (CB_2_) signals *via* G-alpha-s and induces IL-6 and IL-10 cytokine secretion in human primary leukocytes. ACS Pharmacol. Transl. Sci..

[bib67] Shao Z., Yin J., Chapman K., Grzemska M., Clark L., Wang J., Rosenbaum D.M. (2016). High-resolution crystal structure of the human CB1 cannabinoid receptor. Nature.

[bib68] Shimamura T., Shiroishi M., Weyand S., Tsujimoto H., Winter G., Katritch V., Abagyan R., Cherezov V., Liu W., Han G.W. (2011). Structure of the human histamine H1 receptor complex with doxepin. Nature.

[bib69] Shoyama Y., Hirano H., Oda M., Somehara T., Nishioka I. (1975). Cannabichromevarin and Cannabigerovarin, two new propyl homologues of cannabichromene and cannabigerol. Chem. Pharm. Bull..

[bib70] Shoyama Y., Yamauchi T., Nishioka I. (1970). Cannabis. V. Cannabigerolic acid monomethyl ether and cannabinolic acid. Chem. Pharm. Bull..

[bib71] Slipetz D.M., Neill G.P., Favreau L., Dufresne C., Gallant M., Gareau Y., Guay D., Labelle M., Metters K.M. (1995). Activation of the human peripheral cannabinoid receptor results in inhibition of adenylyl cyclase. Mol. Pharmacol..

[bib72] Soethoudt M., Grether U., Fingerle J., Grim T.W., Fezza F., De Petrocellis L., Ullmer C., Rothenhäusler B., Perret C., Van Gils N. (2017). Cannabinoid CB_2_ receptor ligand profiling reveals biased signalling and off-target activity. Nat. Commun..

[bib73] Stella N., Schweitzer P., Piomelli D. (1997). A second endogenous cannabinoid that modulates long-term potentiation. Nature.

[bib74] Sugiura T., Kondo S., Sukagawa A., Nakane S., Shinoda A., Itoh K., Yamashita A., Waku K. (1995). 2-Arachidonoylglycerol: a possible endogenous cannabinoid receptor ligand in brain. Biochem. Biophys. Res. Commun..

[bib75] Tham M., Yilmaz O., Alaverdashvili M., Kelly M.E.M., Denovan-Wright E.M., Laprairie R.B. (2019). Allosteric and orthosteric pharmacology of cannabidiol and cannabidiol-dimethylheptyl at the type 1 and type 2 cannabinoid receptors. Br. J. Pharmacol..

[bib76] Thomas A., Baillie G.L., Phillips A.M., Razdan R.K., Ross R.A., Pertwee R.G. (2007). Cannabidiol displays unexpectedly high potency as an antagonist of CB_1_ and CB_2_ receptor agonists *in vitro*. Br. J. Pharmacol..

[bib77] Turner S.E., Williams C.M., Iversen L., Whalley B.J., Kinghorn A.D., Falk H., Gibbons S., Kobayashi J. (2017). Molecular pharmacology of phytocannabinoids. Phytocannabinoids: Unraveling the Complex Chemistry and Pharmacology of *Cannabis Sativa*.

[bib78] Tuteja N. (2009). Signaling through G protein coupled receptors. Plant Signal. Behav..

[bib79] Vijayakumar S., Manogar P., Prabhu S., Pugazhenthi M., Praseetha P.K. (2019). A pharmacoinformatic approach on Cannabinoid receptor 2 (CB_2_) and different small molecules: homology modelling, molecular docking, MD simulations, drug designing and ADME analysis. Comput. Biol. Chem..

[bib80] Vollner L., Bieniek D., Korte F. (1969). Haschisch XX: cannabidivarin, ein neuer Haschisch-Inhaltsstoff. Tetrahedron Lett..

[bib81] Vriend G. (1990). WHAT IF: a molecular modeling and drug design program. J. Mol. Graph..

[bib82] Wang C., Wu H., Katritch V., Han G.W., Huang X.-P., Liu W., Siu F.Y., Roth B.L., Cherezov V., Stevens R.C. (2013). Structure of the human smoothened receptor bound to an antitumour agent. Nature.

[bib83] Warne T., Moukhametzianov R., Baker J.G., Nehmé R., Edwards P.C., Leslie A.G.W., Schertler G.F.X., Tate C.G. (2011). The structural basis for agonist and partial agonist action on a β1-adrenergic receptor. Nature.

[bib84] Weis W.I., Kobilka B.K. (2014). The molecular basis of G protein–coupled receptor activation. Annu. Rev. Biochem..

[bib85] Wess J. (1997). G-protein-coupled receptors: molecular mechanisms involved in receptor activation and selectivity of G-protein recognition. FASEB J..

[bib86] Wu B., Chien E.Y.T., Mol C.D., Fenalti G., Liu W., Katritch V., Abagyan R., Brooun A., Wells P., Bi F.C. (2010). Structures of the CXCR4 chemokine GPCR with small-molecule and cyclic peptide antagonists. Science.

[bib87] Xing C., Zhuang Y., Xu T.-H., Feng Z., Zhou X.E., Chen M., Wang L., Meng X., Xue Y., Wang J. (2020). Cryo-EM structure of the human cannabinoid receptor CB2-Gi signaling complex. Cell.

